# Graphene-based plasmonic metamaterial for terahertz laser transistors

**DOI:** 10.1515/nanoph-2021-0651

**Published:** 2022-02-02

**Authors:** Taiichi Otsuji, Stephane Albon Boubanga-Tombet, Akira Satou, Deepika Yadav, Hirokazu Fukidome, Takayuki Watanabe, Tetsuya Suemitsu, Alexander A. Dubinov, Vyacheslav V. Popov, Wojciech Knap, Valentin Kachorovskii, Koichi Narahara, Maxim Ryzhii, Vladimir Mitin, Michael S. Shur, Victor Ryzhii

**Affiliations:** Research Institute of Electrical Communication, Tohoku University, Sendai 9808577, Japan; Center for Innovative Integrated Electronic Systems, Tohoku University, Sendai 9808572, Japan; Institute for Physics of Microstructures, Russian Academy of Sciences, Lobachevsky State University of Nizhny Novgorod, Nizhny Novgorod 603950, Russia; Kotelnikov Institute of Radio Engineering and Electronics (Saratov Branch), Russian Academy of Sciences, Saratov 410019, Russia; CENTERA Laboratories, Institute of High Pressure Physics, Warsaw PAS 01142, Poland; Laboratory Charles Coulomb, University of Montpellier and CNRS, Montpellier F-34095, France; Ioffe Institute, St. Petersburg 194021, Russia; Department of Electrical and Electronic Engineering, Kanagawa Institute of Technology, Atsugi, Kanagawa 243-0292, Japan; Department of Computer Science and Engineering, University of Aizu, Aizu-Wakamatsu 965-8580, Japan; Department of Electrical Engineering, University at Buffalo, SUNY, Buffalo, NY 14260, USA; Department of Electrical, Computer, and Systems Engineering, Rensselaer Polytechnic Institute, Troy, NY 12180, USA; Mokerov Institute of Ultra-High Frequency Semiconductor Electronics, RAS, Moscow 117105, Russia

**Keywords:** current injection pumping, Dirac plasmons, distributed-feedback, dual-grating-gate, graphene, instability, lasers, metamaterial, parity- and time-reversal symmetry, terahertz

## Abstract

This paper reviews recent advances in the research and development of graphene-based plasmonic metamaterials for terahertz (THz) laser transistors. The authors’ theoretical discovery on THz laser transistors in 2007 was realized as a distributed-feedback dual-gate graphene-channel field-effect transistor (DFB-DG-GFET) in 2018, demonstrating ∼0.1 µW single-mode emission at 5.2 THz and ∼80 µW amplified spontaneous 1–7.6 THz emission at 100 K. To realize room-temperature, dry-cell-battery operating intense THz lasing with fast direct modulation, various approaches based on graphene plasmonic metamaterials are investigated and introduced as real device implementations, including (i) replacement of the laser photonic cavity with plasmonic cavity enormously improving the THz photon field confinement with larger gain overlapping, (ii) introduction of THz amplification of stimulated emission via current-driven graphene Dirac plasmons (GDPs), and (iii) controlling the parity and time-reversal symmetry of GDPs enabling ultrafast direct gain-switch modulation. Possible real device structures and design constraints are discussed and addressed toward coherent light sources applicable to future 6G- and 7G-class THz wireless communication systems.

## 1 Introduction

Terahertz (THz) electromagnetic waves are important, but still unexplored frequency range bridging radio and light waves. Compact, high power, and room temperature coherent THz light sources are in high demand to realize vast applications of THz waves [1], [2], [3], [4], [5], such as safe and secure non-destructive imaging [6], [7], [8], [9], [10], biomedical inspection [11], [12], [13], as well as ultra-broadband, high-speed wireless communications [14], [15], [16]. In particular, the next-generation ultra-high-speed, ultra-broadband 6G and 7G THz wireless communication systems are big stars with hopes to realize ubiquitous, sustainable, and resilient super-smart society [4, 15, 16]. To make such systems possible, the realization of room-temperature and dry-cell-battery operating, intense, THz lasers with the capability of ultrafast modulation around 100 Gbit/s are the mandatory conditions [15, 16]. THz quantum cascade lasers (QCLs) [5, 17], [18], [19], [20], [21], [22] have improved their performances and reached operating temperatures up to 250 K [5] but still suffer from the phonon decoherence [5, 21]. A unique technique of difference-frequency generation in a single mid-infrared (mid-IR) QCL serving dual laser cavities and lasing frequencies now enables room-temperature THz lasing [23]. However, it still under needs improvement of spectral purity and stability, also due to the phonon decoherence as any other photonic devices do. Resonant tunneling diode (RTD) oscillators [24, 25], on the other hand, have increased their maximal oscillating frequencies to closely 2 THz [24], but substantially limit their output power inversely proportional to the square to the third power of frequency due to the electron-transit-time effect [25] as many other electron devices do. Therefore, the introduction of new physics and/or new materials is necessary to break through all these substantial limitations. Graphene has attracted attention, as one of the most promising materials, owing to its gapless and linear energy spectrum and massless Dirac fermions giving rise to many superior carrier-transport, optical, and plasmonic properties [26], [27], [28], [29], [30], [31].

Optical and/or injection pumping of graphene can induce negative-dynamic conductivity in the THz spectral range enabling new types of THz lasers [32], [33], [34], [35]. In the graphene structures with p-i-n junctions, the injected electrons and holes have relatively low energies compared with those created by optical pumping. Therefore, the effect of carrier cooling can be quite pronounced, providing a significant advantage of the injection pumping in the realization of graphene THz lasers [35, 36]. The authors’ theoretical discovery on THz laser transistors in 2007 was realized in a forward-biased graphene structure with a lateral p-i-n junction in a distributed-feedback dual-gate graphene-channel field-effect transistor (DFB-DG-GFET) and experimentally demonstrated a world-first ∼0.1 µW single-mode emission at 5.2 THz and ∼80 µW amplified spontaneous 1–7.6 THz emission, both at 100 K in 2018 [37]. The relatively weak output intensities and relatively low lasing threshold temperature are major DFB-DG-GFET subjects to be improved.

This pioneering work, research, and study advanced the graphene THz laser transistors. To realize room-temperature, dry-cell-battery operating intense THz lasing with fast direct modulation, various approaches based on graphene plasmonic metamaterials have been investigated and introduced as promising real device implementations. They include (i) replacement of the laser photonic cavity with plasmonic cavity enormously improving the THz photon field confinement with larger gain overlapping [38], [39], [40], [41], (ii) excitation of carrier-population-inverted graphene surface plasmon polaritons, which we call it graphene Dirac plasmon polariton (GDPP) hereafter [42], [43], [44], [45], [46], [47], (iii) introduction of THz amplification of stimulated emission via instabilities of current-driven graphene Dirac plasmons (GDPs) [48, 49], and (iv) an unprecedented trend on controlling the parity and time-reversal symmetry of GDPs enabling ultrafast direct gain-switch modulation [50], [51], [52], [53], [54]. This paper reviews these recent advances of graphene-based plasmonic metamaterial for THz laser transistors. Possible real device structures and design constraints are also discussed and addressed toward coherent light sources applicable to future 6G- and 7G-class THz wireless communication systems.

## 2 Review of the early works on current-injection graphene THz laser transistors

### 2.1 Theory of THz gain in graphene under current-injection pumping

The real part of the dynamic conductivity Re*σ*(*ω*) in graphene under current-injection pumping is given by the sum of the intraband Drude-like component Reσintra(ω) and the interband transition-related component Reσinter(ω) [32, 35, 37]:

(1) Reσ(ω)=Reσintra(ω)+Reσinter(ω),

(2) Reσintra(ω)≈(ln 2+ϵF2kBT)e2πℏ2kBTτ(1+ω2τ2),

(3) Reσinter(ω)=e24ℏ[f(−ℏω/2)−f(ℏω/2)]≈e22ℏexp(eVd−2eVFVg2kBT)sinh(ℏω−eVd2kBT),

(4) $$\epsilon_{F}\approx \hbar v_{F}\sqrt{\frac{\kappa V_{g}}{4ed}}\equiv e\sqrt{V_{F}V_{g},}$$

where $V_{F}=(\hbar^{2}v_{F}^{2}\varepsilon /4e^{3}d)$ , *e* is the elementary charge, f(ϵ) is the Fermi–Dirac distribution function for electrons, ℏ is the reduced Planck’s constant, $k_{B}$ is the Boltzmann constant, *T* is the carrier temperature, τ is the momentum relaxation time, $v_{F}$ is the Fermi velocity, *κ* is the permittivity of the gate dielectric layer, *d* is the gate-dielectric layer thickness, and $V_{d}$ is the applied drain-source bias voltage. Reσintra(ω) is always positive and monotonically decreasing with increasing *ω* as $\frac{1}{1+\omega^{2}\tau^{2}}$ . The roll-off frequency is $\omega_{\text{roll}-\text{off}}∼1/\tau$ . Reσinter(ω) has transition at around $\hbar \omega ∼2\epsilon_{F},$ and reaches the upper plateau approaching $e^{2}/4\hbar$ at high frequencies $\hbar \omega \gg 2\epsilon_{F}$ and the lower plateau approaching 0 at low frequencies $\hbar \omega \ll 2\epsilon_{F}$ when the carrier populations are equilibrated. On the contrary, when the carrier populations are inverted by the carrier-injection pumping at a certain value of $V_{d}$ , the minimal plateau of Reσinter(ω) may shift to a negative level (as low as $e^{2}/4\hbar$ (∼2.3%)). As a consequence, if $\hbar \omega_{\text{roll}-\text{off}}\ll 2\epsilon_{F}$ there exists a certain frequency range in which the Reσ(ω) is negative. How widely and deeply the conductivity goes negative at a given $V_{d}$ depends directly on τ and *T*. A longer τ lowers the roll-off frequency and the conductivity values at higher cutoff frequencies (ωτ≫1). A lower *T* helps increase the population inversion to higher levels and sharpens the transition of Reσinter(ω) at around $\hbar \omega ∼2\epsilon_{F}$ , enlarging the upper cutoff frequency of the negative conductivity (gain) spectrum. To obtain a rather wide gain spectrum in the THz range, τ should be as long as possible (at least picoseconds) and *T* should be as low as possible. The roll-off frequency $\omega_{\text{roll}-\text{off}}\left(∼1/\tau \right)$ directly affects the lower cutoff frequency of the gain spectra. In a real situation with *τ* values on the order of picoseconds, which are well obtainable in high-quality graphene even at room temperatures, the possible gain-spectral windows open well over the THz frequency range.

### 2.2 Experimental results and critical issues

**Figure 1:**
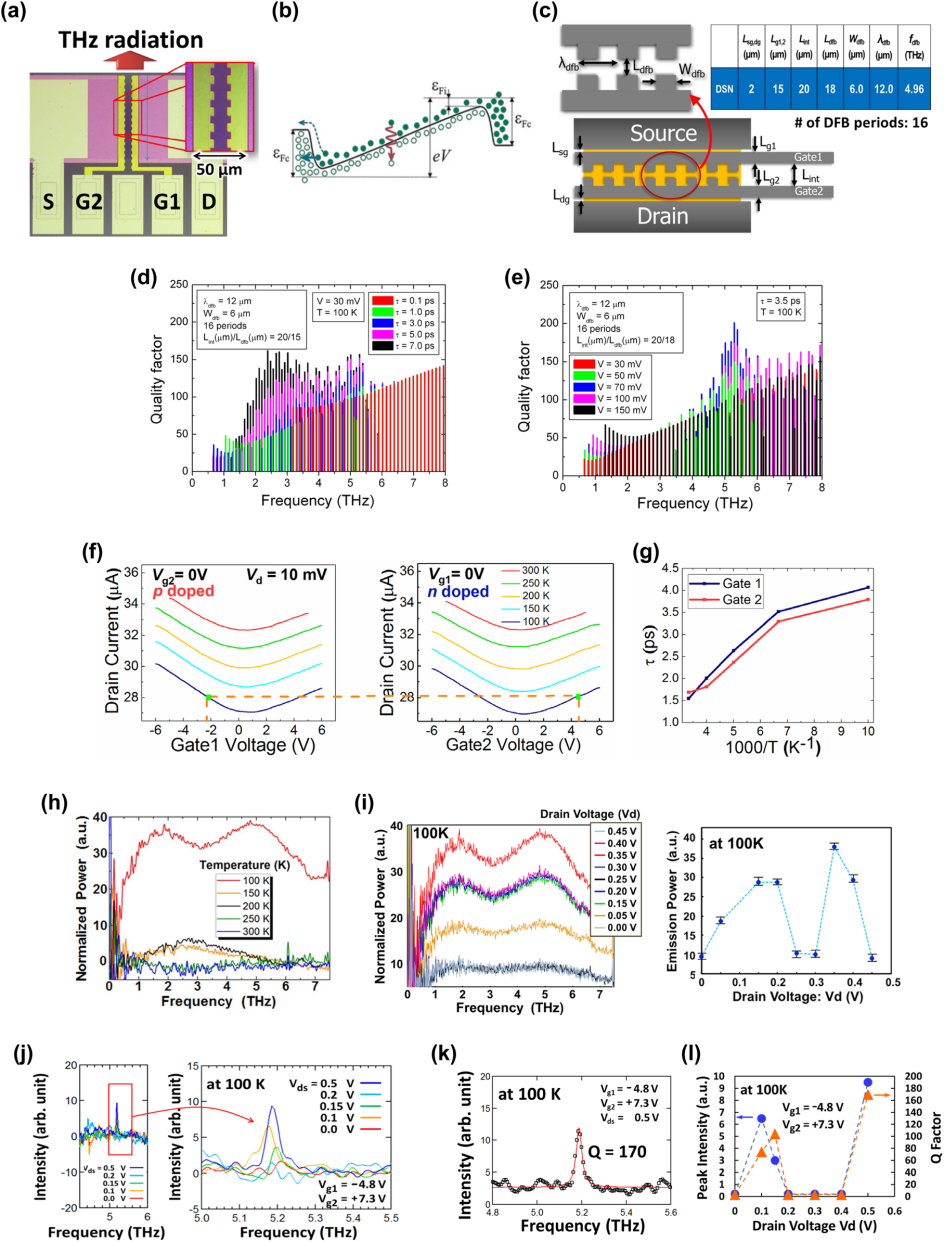
First experimental demonstration for THz single-mode emission and THz amplified broadband spontaneous emission (ASE) in DFB-DG-GFETs.(a) Microscope top-view image of the device. (b) Energy band diagram for carrier population inversion under electrical pumping. (c) DFB cavity structure and physical design parameters. (d) Numerically simulated frequency spectral dependencies of the *Q* factors for different τ values. The DFB cavity design parameters are denoted in the figure and the applied drain-source bias *V*_d_ was assumed to be 30 mV. Adapted with permission from ref. [37]. Copyright 2018, the Authors, published by De Gruyter. (e) Numerically simulated frequency spectral dependencies of the *Q* factors for different *V*_d_ values. The DFB cavity design parameters are denoted in the figure and the applied drain-source bias τ value was assumed to be 3.5 ps. Adapted with permission from ref. [37]. Copyright 2018, the Authors, published by De Gruyter. (f) Measured ambipolar current–voltage characteristics of device 1 for G1 (left) and G2 (right) voltages with *V*_d_ = 10 mV. The square dots are typical points for symmetric electron/hole injections. Adapted with permission from ref. [37]. Copyright 2018, the Authors, published by De Gruyter. (g) Temperature dependence of the extracted carrier momentum relaxation time (*τ*) for device 1. Adapted with permission from ref. [37]. Copyright 2018, the Authors, published by De Gruyter. (h) Observed emission spectra of device 1 at different temperatures exhibiting the THz amplified spontaneous broadband emission at 100 K below a threshold temperature between 100 and 150 K. Adapted with permission from ref. [37]. Copyright 2018, the Authors, published by De Gruyter. (i) Observed emission spectra of device 1 at 100 K for different drain voltages *V*_d_ (left) and *V*_d_ dependence of their emission powers (right). Adapted with permission from ref. [37]. Copyright 2018, the Authors, published by De Gruyter. (j) Observed emission spectra of device 2 at 100 K for different drain voltages *V*_d_, demonstrating a single-mode 5.2 THz emission under distinctive *V*_d_ conditions. Adapted with permission from ref. [37]. Copyright 2018, the Authors, published by De Gruyter. (k) The maximal single-mode emission spectrum of device 2 at *V*_d_ = 0.5 V fitted to a Lorentzian curve with a *Q* factor of 170 (an equivalent linewidth of 30.6 GHz). Adapted with permission from ref. [37]. Copyright 2018, the Authors, published by De Gruyter. (l) *V*_d_ dependencies of the peak emission intensities and *Q* factors.

Epitaxial graphene was synthesized by the thermal decomposition of a C-face 4H-SiC substrate [37]. The Raman spectrum confirmed a high-quality, few-layers non-Bernal stacked graphene. The GFET was fabricated using standard photolithography and a gate stack with a SiN dielectric layer (see Figure 1(a)–(c)), providing an excellent intrinsic field-effect mobility exceeding 100,000 cm^2^/Vs at 300 K at the maximal transconductance [37]. A pair of teeth-brush-shaped gate electrodes was patterned to form a DFB photonic laser cavity in which the active gain area and corresponding gain coefficient were spatially modulated (see Figure 1(c)–(e)) [37]. The key design parameters for the DFB cavity are the grating period *Λ,* the effective refractive index $n_{eff}$ the Bragg wavelength $\lambda_{dfb},$ the principal mode frequency $f_{p}$ , and the modulation index *I*_mod_ = *L*_int_/*L*_dfb_ (*L*_int_ and *L*_dfb_ are the distance between the dual gates at the wider and narrower region, respectively, see Figure 1(c)). The values of these parameters were set to be *Λ* = 16, $n_{eff}=2.52$ , $\lambda_{dfb}=12\mathrm{\mu m}$ , $f_{p}=4.96\text{ THz}$ , identically, and *I*_mod_ = 20/15 for device 1 and *I*_mod_ = 20/18 for device 2, respectively. As shown in Figure 1(d) and (e) the frequency response of the DFB cavity is too sensitive to the carrier transport property of momentum relaxation time *τ*; rather modestly low *τ* values give rise to single-mode resonant responses at the fundamental mode frequency of ∼5.0 THz, whereas longer *τ* values screen the resonant response and give rise to broadband ‘cavity-less’ responses. Such a peculiar DFB cavity response was identified to be due to poor gain overlapping as discussed later.

We carried experiments on the two devices, device 1 and device 2. The fabricated DFB-DG-GFETs exhibited an ambipolar behavior in the current-to-gate voltages (*V*_g1_, *V*_g2_) characteristics. Figure 1(f) illustrates typical ambipolar properties of device 1. With decreasing temperature, the thermionic current reduces i.e. the level of drain current decreases but the transconductance (*g*_m_), the slope of the ambipolar curves, increases reflecting the longer *τ* values at lower temperatures. The *τ* values for these two devices, which were extracted from the slope of the ambipolar current–voltage curves, had different values: device 1 having longer values (ranging from 1.5 to 4 ps at temperatures 300–100 K as plotted in Figure 1(g)) than those for device 2 (ranging from 0.8 to 2 ps at temperatures 300–100 K) [37]. One can expect negative conductivities to be obtained more easily at lower temperatures, enabling enhanced THz emission.

The THz emission was measured at temperatures from 300 K down to 100 K using a Fourier-transform spectrometer with a 4.2 K-cooled Si bolometer. The background blackbody radiation was observed under the zero-bias condition and was subtracted from the radiation observed under biased conditions. Device 1 exhibited a rather strong emission at 100 K, stronger than that at higher temperatures from 150 to 300 K as shown in Figure 1(h). The emission was observed in 1–7.6 THz range when *V*_d_ was forward-biased to a certain level under symmetric electron and hole injection conditions (*V*_g2_ = 4.56 V, *V*_g1_ = −2.58 V) leading to the carrier population inversion [37]. The first peak at ∼2 THz came from the original gain spectral profile [37], whereas the second peak at ∼5 THz coincided with the fundamental DFB mode frequency. The substrate-thickness-dependent THz photon field distribution could not meet the maximal available gain-overlapping condition as is discussed later. As a result, the device failed the single-mode lasing and exhibited only the amplified spontaneous broadband THz emission. Apart from temperature-dependent spontaneous THz emission, the device also exhibited double-threshold-like behavior with respect to the current-injection pumping levels corresponding to the applied *V*_d_ levels with the maximal emission intensity ∼80 μW as shown in Figure 1(i). Such a double-threshold-like behavior is considered to be due to the carrier overcooling effect in weakly pumped graphene [34, 35, 37]. The authors theoretically revealed the occurrence of the carrier overcooling at low pumping levels due to electron kinetic inertia enabling excess heat energy transfer to the lattice phonons. This may help promote the carrier population inversion and spontaneous THz emission [34, 35, 37]. With increased pumping, the carrier overcooling weakens but the level of population inversion increases. Such competition between the carrier overcooling and population inversion may result in the observed double-threshold-like behavior [37].

The experiment was also conducted for device 2, having similar design geometries and fabricated on the identical wafer. The observed emission spectra at 100 K exhibited a rather sharp single-mode emission at 5.2 THz when *V*_d_ was positively applied to a certain level under symmetric electron and hole injection conditions (see Figure 1(j)) [37]. The emission spectra with the highest peak intensity at *V*_d_ = 0.5 V could fit the Lorentzian curve with the *Q* factor of 170 (a linewidth of 30.6 GHz) (see Figure 1(k)), which agrees well with the simulated results shown in Figure 1(e) [37]. As was observed in the case of the aforementioned amplified broadband spontaneous emission in device 1, the single-mode emission in device 2 also exhibited a non-monotonic double-threshold-like behavior with respect to the applied *V*_d_ with the highest intensity ∼0.1 μW as shown in Figure 1(k). Spectral narrowing and increase in *Q* values with increasing *V*_d_ around the double threshold levels were also observed as shown in Figure 1(k).

The fundamental limit on poor output intensity is not primarily attributed to the quantum-mechanical limit of the nature of graphene properties of the interband-absorption coefficient of maximal $e^{2}/4\hbar$ (2.3%) per monolayer given by Eq. (1). It can be mainly attributed to poor gain overlapping factor due to insufficient THz photon field confinement with an inefficient ‘photonic’ laser cavity design as is discussed in the next section. The relatively low lasing threshold temperature is thought to be attributed to the mixture of the poor gain overlapping and non-ideal carrier transport with scattering due to the imperfection of graphene crystallographic quality. At first, to make single-mode lasing, one needs to implement a pertinent high-*Q* (Quality factor) laser cavity structure accommodating the gain medium of graphene under carrier injection pumping. Furthermore, to break through the $e^{2}/4\hbar$ (2.3%) limit on quantum efficiency one has to take a different route, for example, introducing the graphene plasmonic metamaterial design. This is the most promising solution, which is described in various aspects in the next section.

## 3 Graphene plasmonic metamaterial design for performance improvements

### 3.1 Plasmonic laser cavity and plasmonic lasing

**Figure 2:**
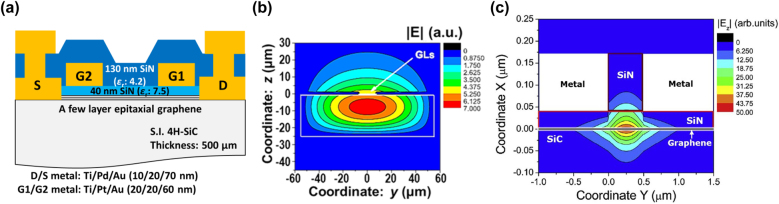
Numerically simulated THz in-plane (*y*-axial component) field distributions in DFB-DG-GFETs with photonic versus plasmonic cavities. (a) Device cross-sectional structure. (b) Cross-sectional contour map of the simulated THz photon field distribution of in-plane electric field component in a photonic DFB cavity structure when the SiC substrate is thinned to a 25 µm thickness (GLs: graphene layers). Adapted with permission from ref. [38]. Copyright 2011, IOP Publishing Ltd. [38]. (c) Cross-sectional contour map of the simulated THz surface plasmon-polariton field distribution of in-plane electric field component in a plasmonic DFB cavity structure. Adapted with permission from ref. [41]. Copyright 2021, Association of Polish Electrical Engineers (SEP), published by Elsevier B.V.

Figure 2 draws (a) the cross-sectional device structure and (b) numerically simulated cross-sectional THz photon field distribution of its in-plane electric field intensity component even when the SiC substrate is thinned down to a 25 µm thickness. Due to the effect of the high-permittivity SiC substrate underneath the graphene (situated the top surface of the SiC substrate) the field of spontaneously emitted THz photons from the population-inverted graphene channel layer sank into the SiC substrate region, resulting in poor field overlapping with the graphene region and a poor gain overlapping factor even if the SiC substrate thickness was thinned down to 25 µm as shown in Figure 2(b). This results in a poor THz gain coefficient so that the laser cavity does not work well. After resolving this poor gain-overlapping issue, the fundamental quantum-mechanical limit of interband-absorption coefficient of maximal 2.3% per monolayer is the next performance-limiting factor.

To resolve such a critical issue of poor gain overlapping, confinement of the THz photon field close to the graphene region is the key, and the introduction of a graphene plasmonic laser cavity can make it possible. GDPs are the quanta of collected charge density waves of graphene Dirac fermions whose group velocity is by two orders of magnitude lower than that of light in a vacuum. Such a slow-wave nature gives rise to the spatial confinement of the plasmon field that is by two orders of magnitude higher than that of the photon field. Figure 2(c) shows typical simulated results illustrating how the plasmonic cavity work for the THz photon field confinement. One can see that the GDP field is strongly confined to the proximity of the active graphene region.

**Figure 3:**
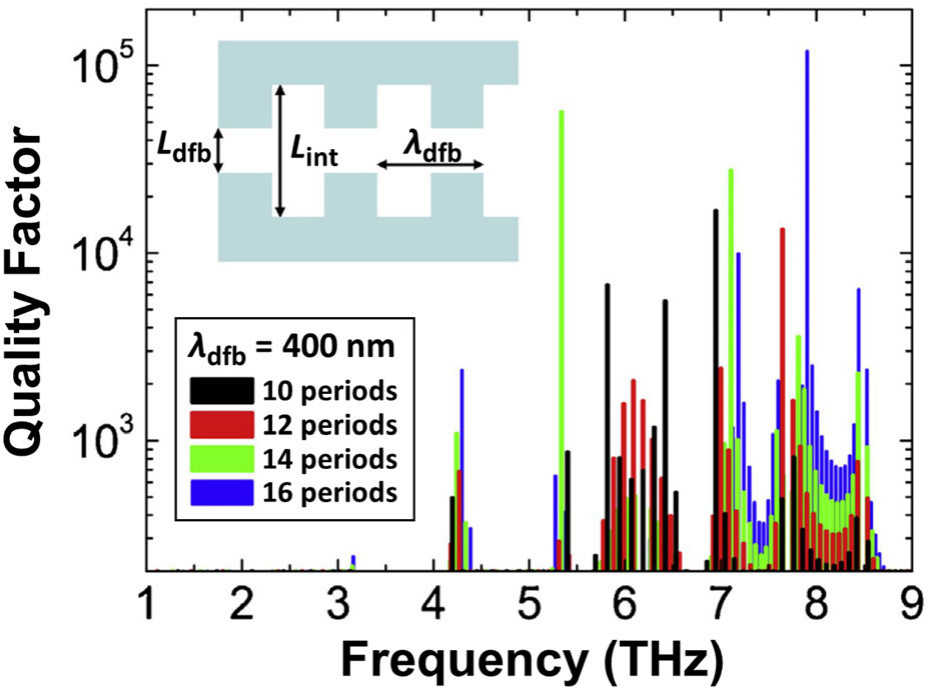
Numerically simulated frequency spectral dependencies of the quality factors for different number of periods. The inset shows the top view of a plasmonic DFB cavity structure with key design parameters of *λ*_dfb_ = 400 nm, *L*_dfb_ = 200 nm, and *L*_int_ = 500 nm. Adapted with permission from ref. [41]. Copyright 2021, Association of Polish Electrical Engineers (SEP), published by Elsevier B.V.

Figure 3 plots numerically simulated frequency spectral dependencies of the Q factors for different quantity of periods at the DFB cavity parameters with *λ*_dfb_ = 400 nm, *L*_dfb_ = 200 nm, and *L*_int_ = 500 nm (the inset shows the scheme of a plasmonic DFB cavity structure). Compared to the cases for photonic DFB cavities shown in Figure 1(c), the DFB period *λ*_dfb_ is slunk down by more than one order of magnitude due to the slow-wave nature of the plasmons. This gives rise to enormous spatial field confinement, reflecting on the giant enhancement of the *Q* factor above 10^4^, which is more than two orders of magnitude larger than that for photonic cavities shown in Figure 1(d) and (e). Figure 3 shows frequency spectra with huge *Q* factors are observed even for a smaller number of periods than that for the photonic cavity with 16 periods. The reason for such a giant enhancement on *Q* factors is due to the substantially large values of the GDP effective refractive indices compared to those for the photonic refractive indices. For the same reason, the required dimensions of the plasmonic DFB cavity structure are substantially smaller in comparison with the photonic DFB cavity structure. Closer inspection of the spectra of the *Q*-factor in Figure 3 shows residual multi-mode resonance characteristics besides the main Fourier harmonic modes, which most probably is excited by a rather complex periodic structure of the DFB cavity. Further design improvement is needed to suppress such unwanted residual resonance modes. The experimental verification of the plasmonic DFB cavity effects will be attempted in the near future.

### 3.2 Excitation of GDPPs for plasmonic gain & amplification

**Figure 4:**
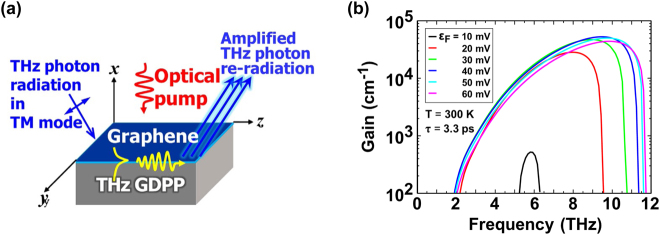
The giant gain enhancement effect of GDPPs on THz stimulated emission in optically pumped monolayer graphene. Adapted with permission from ref. [43]. Copyright 2013, IOP Publishing Ltd. and Deutsche Physikalische Gesellschaft.(a) Schematics of the GDPP excitation. (b) Numerically simulated gain spectra for monolayer population-inverted graphene on SiO_2_/Si substrate at 300 K for different quasi-Fermi energies *ε*_F_ = 10, 20, 30, 40, 50 and 60 meV. Carrier momentum relaxation time *τ* in graphene is *τ* = 3.3 ps. The results demonstrate giant THz gain (negative values of absorption) of the order of 10^4^ cm^−1^.

As is described in Section 2.1, the maximum available gain via interband photon absorption and resultant carrier population inversion is limited to or below $e^{2}/4\hbar$ (2.3%) per monolayer as expressed in Eq. (3). This is the quantum mechanical limit for the THz photons directly interacting with GDFs. Excitation of GDPPs can mediate the interaction between THz photons and graphene Dirac fermions and enormously enhance the interaction between the THz photons and GDFs. When THz electromagnetic radiation impinges on the graphene surface, the GDPPs are excited. The TM (transverse magnetic) mode THz photons are coupled with GDPPs, propagating along the in-plane component direction of the incident THz photon radiation vectors (see Figure 4(a)). In such a situation, the propagation of the SPPs could lead to one of two distinctive scenarios, that is ‘giant loss’ or ‘giant gain’, depending on the conductivity sign. When graphene is normally semi-metallic or semi-conductive with a specific positive (negative) conductivity value, the propagation of the SPPs can drastically enhance the interaction between the THz photons and graphene, resulting in extremely strong ‘absorption’ (‘amplification’) of the incident THz photon energy. This is the case of the ‘giant loss’ (‘giant gain’). The authors’ group first theoretically discovered this phenomenon exhibiting a giant gain coefficient ranging up to the order of 10^4^ cm^−1^ (see Figure 4(b)) [42], and experimentally observed the giant gain enhancement of up to a factor of 50 by using the near-field optical-pump, THz-probe, and optical probe measurement technique in an optically pumped monolayer graphene [43].

The gain spectral profile of population-inverted graphene under optical pumping was more rigorously calculated self-consistently using a random-phase approximation to model the many-body effects of Coulomb-forced intercarrier scattering on the complex-frequency dispersion curves, and was compared with the results obtained using Fermi’s golden rule [44]. The results show that amplification of plasmons is possible under realistic conditions but inevitably competes with ultrafast spontaneous plasmon emission, which is a factor of 5 faster than was previously estimated [44]. The further theoretical investigation was given to dual-grating-gate graphene nanoribbon metasurfaces under current-injection pumping [45], as well as hyperbolic graphene-based multilayered metamaterial structures [46]. The other GDPP excitation scheme with a mixture of optical pumping and dc current injection in graphene metasurfaces was also proposed to obtain THz amplification [47].

It is worth noting again that the excitation of GDPPs may dramatically increase the interaction between incident THz photons and GDFs, resulting in giant enhancement of loss or gain depending on the polarity of the graphene conductivities. This means that, as long as the GDPPs are confined into a gain region, they contribute to increasing amplification. However, once the GDPPs enter into a loss region, they give a strong loss/attenuation. Thus, a transmission-type waveguide structure potentially holds a risk to lose its gain if the GDPP spatial confinement into a gain region is insufficient. In this regard, a cavity structure is appreciated.

**Figure 5:**
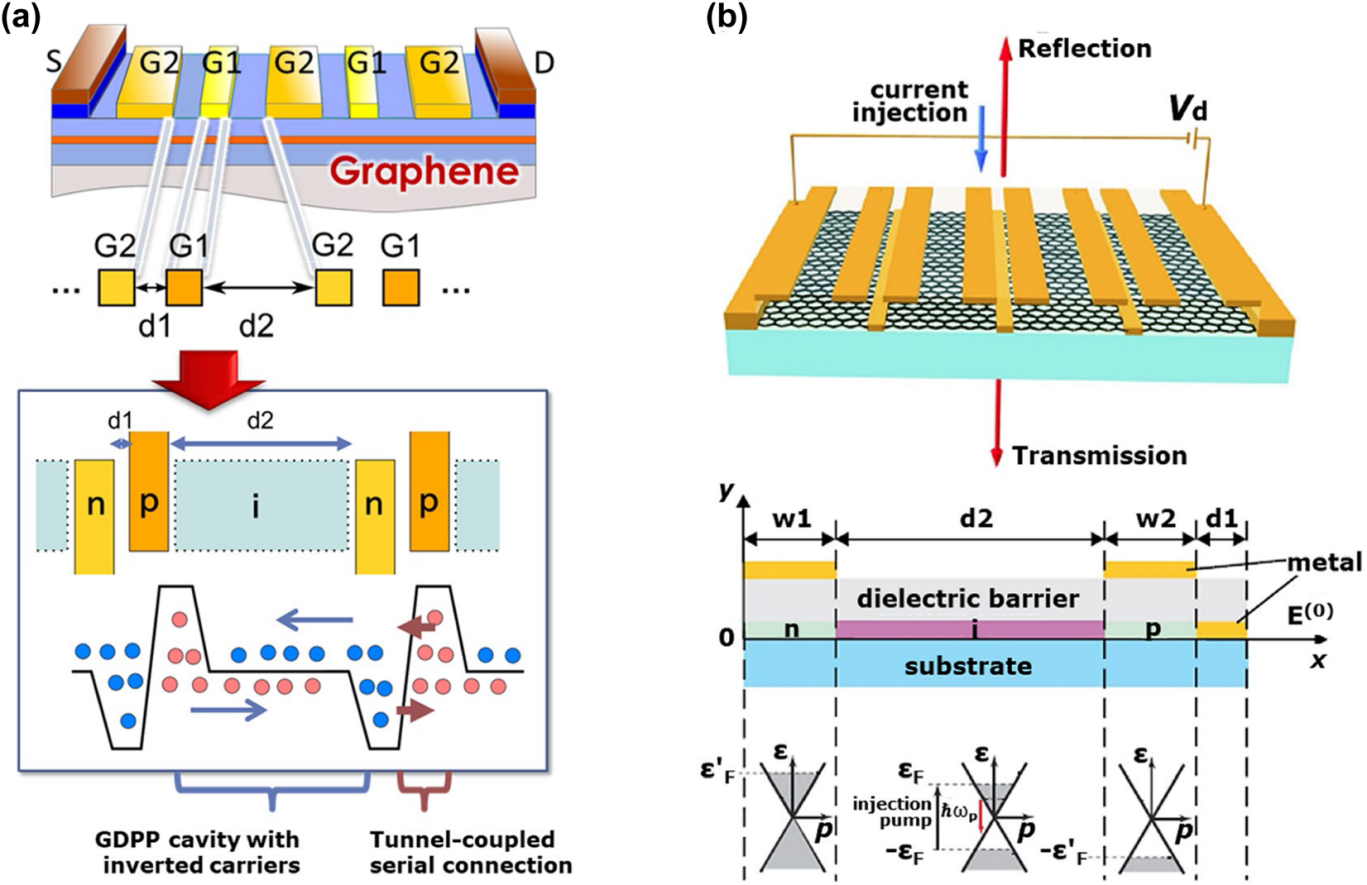
Implementations of the plasmonic THz laser cavities using DGG-GFET structures. (a) Primitive plasmonic THz laser cavity in an asymmetric DGG-GFET structure. The wider d2-spacing ungated region works for the GDPP cavity with inverted carrier and resultant spontaneous THz photon emission under current-injection pumping. The narrower d1-spacing ungated region works for the tunnel-coupled serial connection between adjacent GDPP cavities. Due to the DGG metallic capacitive coupling of the narrower spacing region within the plasmon field overlapping closeness, proximity makes synchronization of uncorrelated deterministic plasmonic lasing among all the DGG channel region giving rise to superradiant plasmonic lasing [40]. (b) A modified plasmonic THz laser cavity in the other asymmetric DGG-GFET structure. Adapted with permission from ref. [45]. Copyright 2017, AIP Publishing Ltd. In-plane floating metal stripes are laid out adding to out-of-plane original asymmetric DGG, but the conceptual mechanism of plasmonic lasing is identical to that shown in Figure 5(a).

There are different variations for the plasmonic cavity structures other than the DFB type described in Section 2.1. The metal grating coupler is one of the most popular structures serving broadband frequency tunability. The dual-grating gate GFET (DGG-GFET) is an improved alternative structure to the DFB-DG-GFET [39, 40, 48]. When the DG electrodes are laid out in an asymmetric geometry where the left- and right-side spacing between adjacent grating fingers differ from each other, the structure works as a plasmonic laser under current-injection pumping (see Figure 5(a)). The wider d2-spacing ungated region forms the GDPP cavity with inverted carrier distribution and resulting spontaneous THz photon emission under optical or electrical pumping [39, 40]. The narrower d1-spacing ungated region provides the tunnel-coupled serial connection between adjacent GDPP cavities [40]. The DGG metallic capacitive coupling of the narrower spacing region within the plasmon field overlapping synchronizes uncorrelated deterministic plasmonic lasing among all the DGG channel regions resulting in the superradiant plasmonic lasing [39, 40]. The theoretical study on the DGG-GFET structures for achieving superradiant plasmonic THz lasing was initiated in the case of optical pumping [39, 40]. Recently this study has been extended to the case of current-injection pumping [45] as well. The periodic DGG structure is the key to defining the resonant modes of the GDPPs. The variations of the DGG-GFET structure include periodic graphene/metallic nanoribbon-patterned channel [39], planar graphene channels with the DGG electrodes beyond the gate insulation layer [40], and/or modified asymmetric DGG-GFETs as shown in Figure 5(b) [45]. In Figure 5(b), in-plane floating metal stripes are laid out adding to out-of-plane original asymmetric DGG, but the conceptual mechanism of plasmonic lasing is identical to the case shown in Figure 5(a).

**Figure 6:**
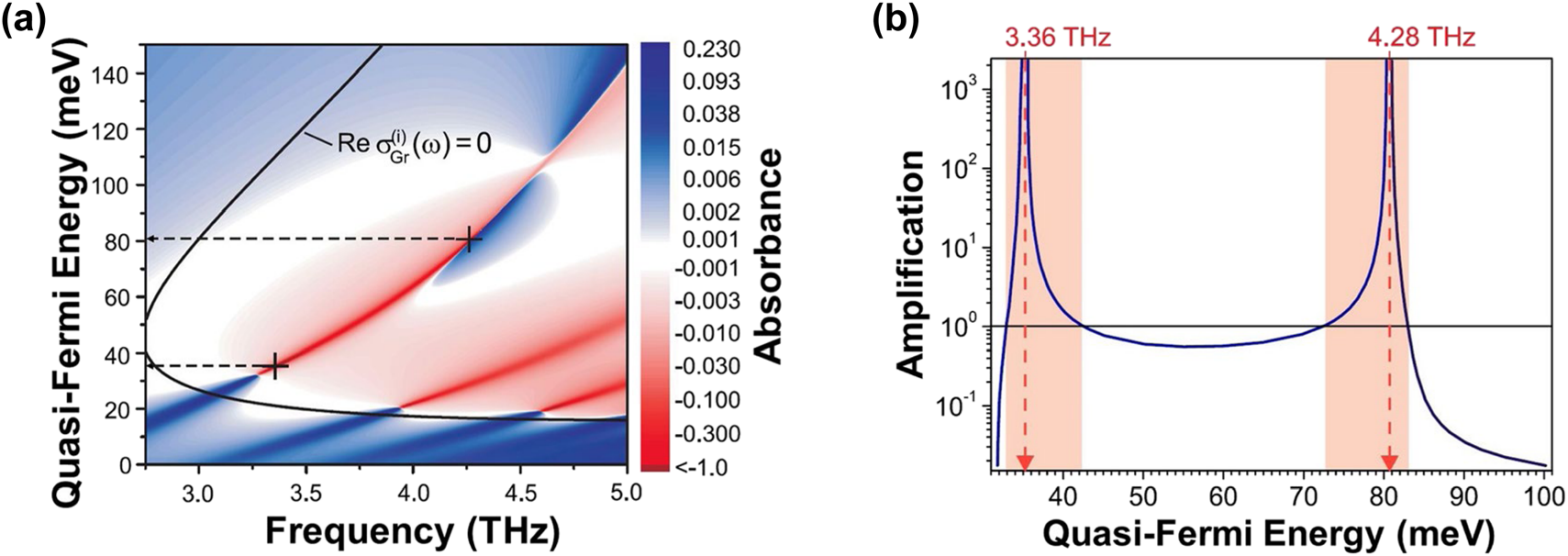
Impact of the plasmonic THz laser cavity in a DGG-GFET for a giant enhancement of resonant amplification gain. (a) Numerically simulated absorbance spectrum for the fundamental and higher-order GDPP resonances in the asymmetric DGG-GFET structure shown in Figure 5(b) as a function of the quasi-Fermi energy $\epsilon_{F}^{\prime }$ and the frequency of the incident THz wave. Adapted with permission from ref. [45]. Copyright 2017, AIP Publishing Ltd. Solid black line corresponds to the transparent regime of the inverted graphene (the real part of the conductivity is zero). The right (left) of this line is the amplification (attenuation) region where graphene works as a gain (loss) medium. The red lines are the routes for the GDPP resonant amplification modes (fundamental to the leftmost, and higher modes to the right). (b) The variation of the amplification coefficient along the lobe of the fundamental plasmon resonance in Figure 6(a) as a function of $\epsilon_{F}^{\prime }$ . Adapted with permission from ref. [45]. Copyright 2017, AIP Publishing Ltd. The values of $\epsilon_{F}^{\prime }$ marked by dashed arrows correspond to the self-excitation regime (plasmon lasing). In the colored regions, the amplified power exceeds that of the incident THz wave.

Figure 6(a) plots the numerically simulated absorbance spectrum for the fundamental and higher-order plasmon resonances in the asymmetric DGG-GFET structure in Figure 5(b) as a function of the quasi-Fermi energy $\epsilon_{F}^{\prime }$ and the frequency of the incident THz wave [45]. Solid black line corresponds to the transparent regime of the inverted graphene (the real part of the conductivity is zero). The right (left) of this line is the amplification (attenuation) region where graphene works as a gain (loss) medium. The red lines are the routes for the GDPP resonant modes (fundamental to the leftmost, and higher modes to the right). Figure 6(b) plots the variation of the amplification coefficient along the lobe of the fundamental plasmon resonance in Figure 6(a) as a function of $\epsilon_{F}^{\prime }$ [45]. The values of $\epsilon_{F}^{\prime }$ marked by dashed arrows correspond to the self-excitation regime (plasmonic lasing). Thanks to the resonant GDPP effect, as seen in Figure 5(b), huge amplification gains of greater than 10^3^ can be expected. In the colored regions, the amplified power exceeds that of the incident THz wave. Experimental verification of the plasmonic THz lasing in these DGG-GFET structures is foreseen.

### 3.3 THz amplification of stimulated emission via current-driven graphene Dirac plasmon instabilities

The generation and amplification of electromagnetic waves by plasmonic instabilities in conventional two-dimensional (2D) electron systems (2DESs) have been actively investigated since 1980. The main idea has been to exploit the radiative decay of grating-coupled 2D plasmonic metasurfaces for the realization of compact tunable solid-state far-infrared devices [56], [57], [58], [59], [60], [61], [62], [63], [64], [65], [66], [67], [68], [69]. These devices present new alternatives to the vacuum devices, such as traveling wave and backward-wave tubes based on the Smith-Purcell effect [68] facing severe difficulties to operate beyond the millimeter-wave region. The rise of graphene and extraordinary stronger light-plasmon coupling in graphene structures compared to any existing massive semiconductor materials can bring revolutionized advancement in this field [27], [28], [29], [30], [31].

**Figure 7:**
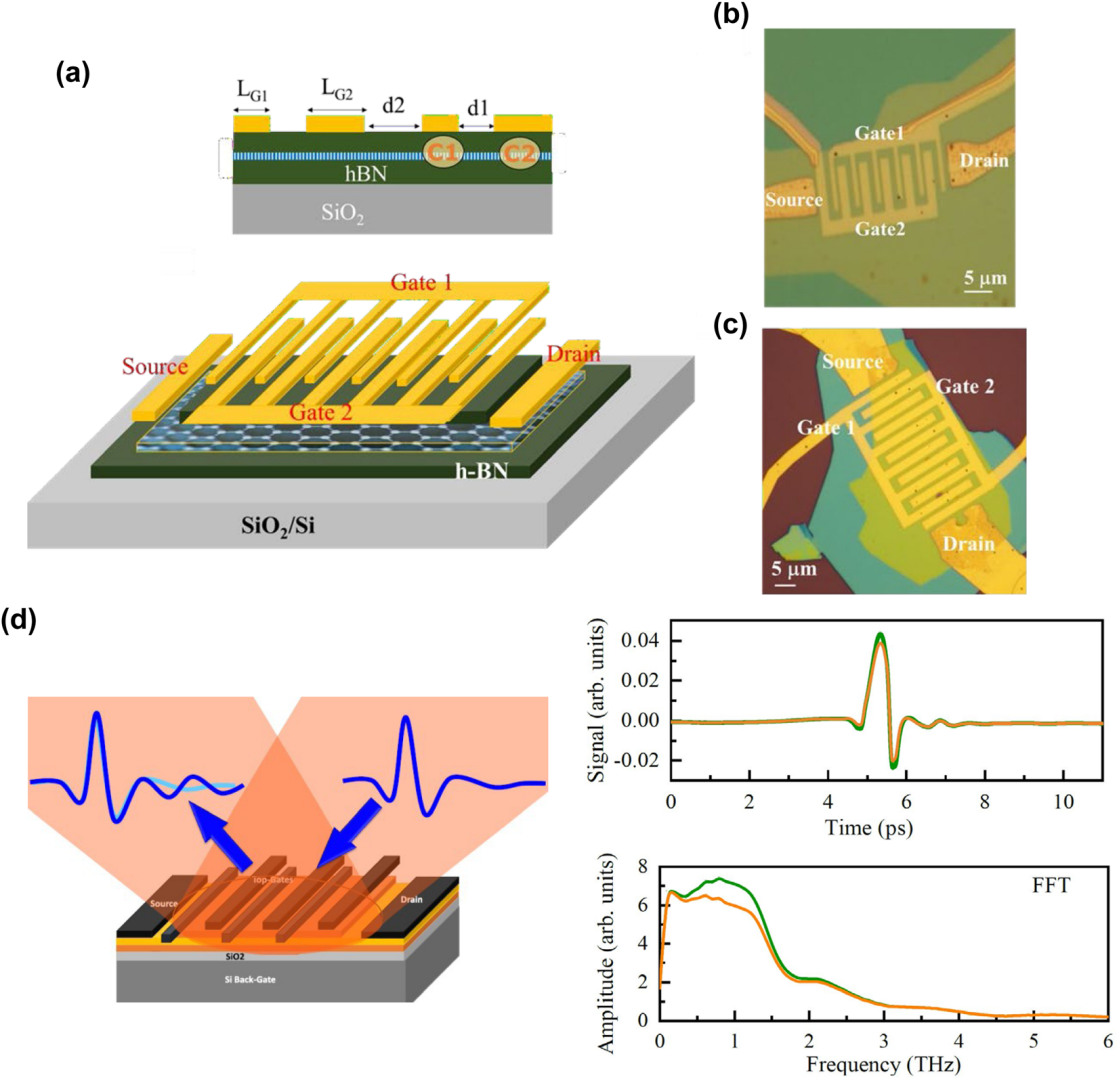
DGG-GFET samples for THz time-domain spectroscopic measurement. (a) Schematic illustrating of the hBN/graphene/hBN heterostructure asymmetric DGG-GFET. Adapted with permission from ref. [49]. Copyright 2021, the Authors, published by Frontiers Media SA. (b) Optical image of an asymmetric DGG-GFET (ADGG-1). Adapted with permission from ref. [49]. Copyright 2021, the Authors, published by Frontiers Media SA. (c) Optical image of the symmetric DGG-GFET (S-DGG). Adapted with permission from ref. [49]. Copyright 2021, the Authors, published by Frontiers Media SA. (d) Experimental geometry of incident THz pulse and transmitted/reflected pulse in an oblique angle (left) and measured temporal waveforms (right upper) and their Fourier spectra (right lower). Orange: incident, green: output. Adapted with permission from ref. [48]. Copyright 2020, the American Physical Society.

We investigated the THz gain and amplification in dc-current-driven metasurfaces promoting the GDP instabilities for use in an efficient tunable absorber, emitter, and amplifier operating at room temperatures. Plasmon modes in the metasurfaces were excited in monolayer graphene on hexagonal boron nitride (hBN) with DGG-GFET structures (two asymmetric DGG (ADGG) types and one symmetric DGG (SDGG) type) as shown in Figure 7(a)–(c) and as summarized in Table 1 [48, 49]. With the applied gate voltages *V*_g1_ and *V*_g2_ each device supports the formation of two different plasmonic cavities (types C1 and C2) with symmetric or asymmetric boundaries below the fingers of Gate 1 and Gate 2 in the DGG device structure. The electron mobilities around 50.000 cm^2^/Vs were obtained from the fabricated devices at room temperatures. DGG-bias-dependent electron density modulation causes spatial complementary modulation of the plasmon and drift velocities. This may cause Doppler-shift (DS) type [58], transit-time-modulation (TTM) type [60], Cherenkov (CK) type [56], and/or plasmonic boom (PB) type instabilities [61]. The former DS type instability may cause under asymmetric cavity boundaries whereas the other types may cause both symmetric and asymmetric boundaries. When the instability-driven gain surpasses the Drude loss, the system yields the net gain, resulting in plasmon self-oscillation at the resonant frequencies of the GDPP cavities. The ADGG works as a broadband antenna that can convert the non-radiative plasma oscillations to radiative THz waves. This, in turn, enables free-running self-oscillatory THz emission of radiation as well as coherent light amplification of stimulated emission to the incident THz waves. We first modeled such a GDPP active metasurfaces and numerically calculate the temporal evolution of the in-plane electric field component after turning on the dc-current flow using self-consistent simulation based on quasi-classical Boltzmann and Poisson equations. The simulation results demonstrated the development of THz self-oscillation at the plasmon mode frequencies depending on the plasmon cavity dimensions and the carrier densities [70].

**Table 1:** Samples specifications.

Device	ADGG-1		ADGG-2		SDGG
Cavity	C1	C2	C1	C2	C1
Biased cavity length (µm)	0.75	1.50	0.5	1.0	2.0
Total channel length (µm)	24.0		26.5		38.5
d1 and d2 (µm)	0.5 and1.0		0.5 and 2.0		1.0
Channel width (µm)	1.325		4.9		3.2
CNP (V)	−0.12		+0.15		−0.10

**Figure 8:**
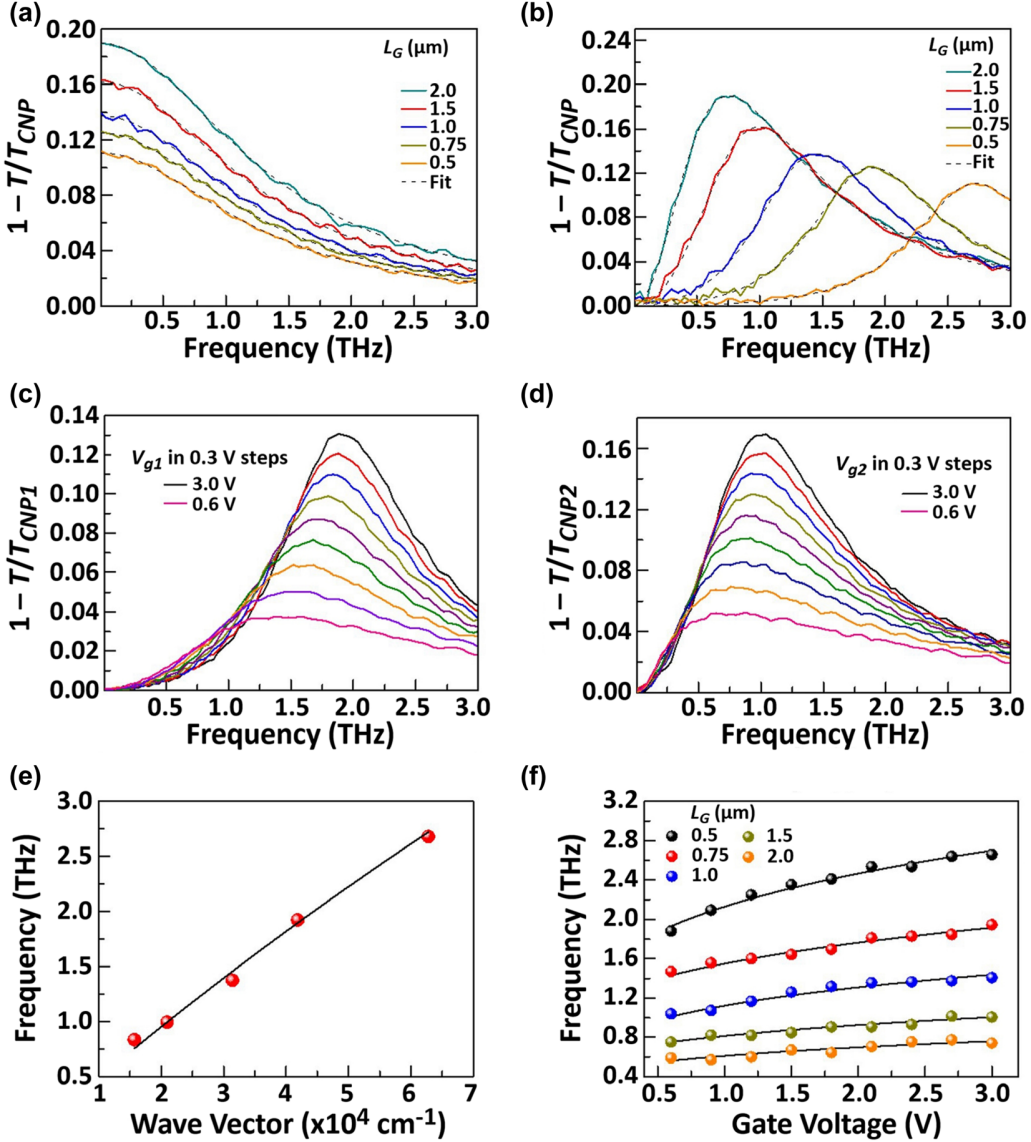
Gate-voltage- and length-dependent extinction spectra of the graphene structures for *V*_d_ = 0 V. Plasmonic cavity length (*L*_G_) dependent extinction spectra of the three devices with incident light polarized parallel (a) and perpendicular (b) to the DGG fingers. The measured line shape is well reproduced by a Drude model fit for parallel polarization (dashed line in (a)) and a damped oscillator model fit for perpendicular polarization (dashed line in (b)). Measured gate voltage-dependent transmission spectra 1−*T*/*T*_CNP_ of the asymmetric device A-DGG1 with incident light polarized perpendicular to the DGG fingers, with biased cavities C1 when sweeping *V*_g1_ while the voltage on the other gate electrode is kept constant at *V*_g2_ = *V*_CNP2_ (c) and biased cavities C2 when sweeping *V*_g2_ while the voltage on the other gate electrode is kept constant at *V*_g1_ = *V*_CNP1_ (d). Scaling laws of graphene plasmon resonance frequency in the three devices as a function of wave vector *q* = *π*/*L*_G_ (e) and gate voltage (f). The solid lines in (e) and (f) are fits to data using the model expressed in Eq. (1). Adapted with permission from ref. [49]. Copyright 2021, the Authors, published by Frontiers Media SA.

The THz time-domain spectroscopy (THz-TDS) was employed to measure the changes in the THz pulses transmitted through the graphene plasmonic cavities of type C1 (C2) when sweeping *V*_g1_ (*V*_g2_) and keeping the voltage on the other gate electrode constant at the charge neutral point (CNP) *V*_g2_ = *V*_CNP2_ (*V*_g1_ = *V*_CNP1_) (see Figure 7(d)). The transmission coefficient at a given (*V*_g1_, *V*_g2_) and *V*_d_ is referred to as *T* while *T*_CNP_ is the transmission coefficient at *V*_g1, g2_ = *V*_CNP_. First, we measured the device response under the zero-*V*_d_ condition. The measured extinction spectra (1−*T*/*T*_CNP_) at *V*_d_ = 0 V exhibited similar tendencies of polarization-sensitive resonant absorptions among the three samples as shown in Figure 8. When the polarization of the incident THz pulse was aligned in parallel to the DGG fingers, the device responded as a non-resonant absorber whose absorption spectrum well traced the Drude free-carrier absorption spectrum (see Figure 8(a)). When the polarization of the incident THz pulse was aligned perpendicular to the DGG fingers, the device responded as resonant absorbers whose peak absorption frequency depended on the GDPP cavity size (see Figure 8(b)). The gate-tunable frequency shift of the resonant absorption was also confirmed (see Figure 8(c) and (d)). Figure 8(e) and (f) plot the dispersion relations with respect to the wave vector (determined by the cavity size) and to the applied gate voltage, respectively. The results corresponded well to the dispersion law for the gated GDPs (linear to the wave vectors and 1/4 power to the gate voltages) [28, 48, 49]. It was concluded that the observed resonant properties are due to the excitation of the GDPPs in the DGG-GFET structures.

**Figure 9:**
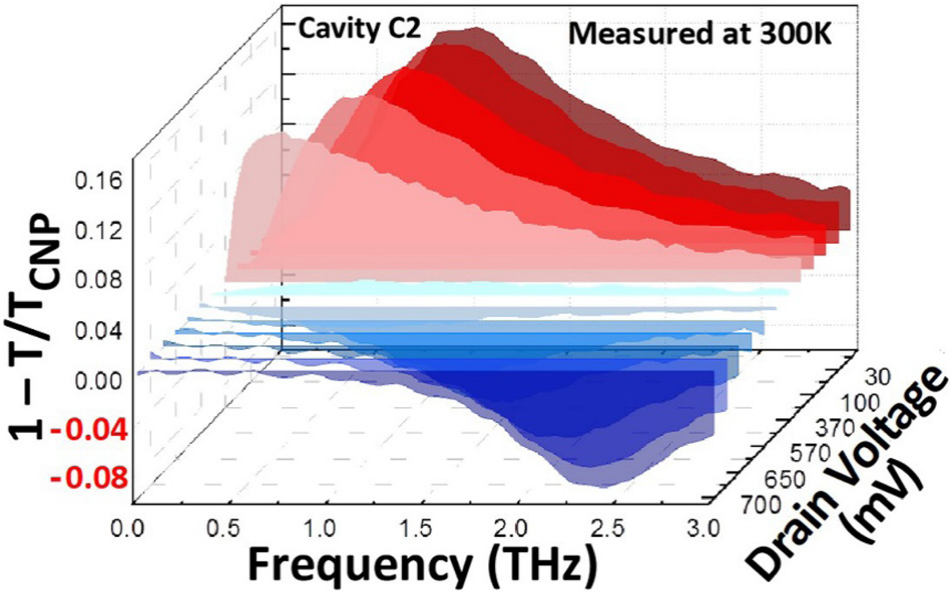
Drain bias dependent extinction spectra of the graphene structures. Spectra measured in cavity C2 of device A-DGG1 for fixed *V*_g2_ − *V*_CNP2_ = 3 V and *V*_g1_ = *V*_CNP1_ when varying *V*_d_. Adapted with permission from ref. [49]. Copyright 2021, the Authors, published by Frontiers Media SA.

Next, we conducted the drain-to-source voltage (*V*_d_) dependent measurements at a fixed (*V*_g1_, *V*_g2_) condition. The most striking features in the measurements arose when we explored the influence of *V*_d_ on the device absorption spectra. Figure 9 depicts the *V*_d_-dependent extinction spectra measured in cavity C2 of the sample A-DGG1 with the electrical doping at *V*_g2_ − *V*_CNP2_ = 3 V, *V*_g1_ = *V*_CNP1_. As *V*_d_ increases the absorption peak clearly exhibited the redshifting along with a noticeable reduction of plasmon resonance strength. Then, the absorption completely vanished when *V*_d_ increased to 370 mV. This indicates that the plasmonic device becomes perfectly transparent to the incoming THz radiation within the entire experimental bandwidth. With increasing *V*_d_ beyond this transparency regime, a negative absorption peak appeared in the extinction spectra from the lower frequency side with a noticeable blue shift. The negative absorption is an indication of a more intense transmitted pulse as compared to the incoming pulse, being referred to as ‘gain’. Throughout all of our experiments, the data from the other asymmetric device (A-DGG2) were quite similar to those shown in Figure 9 [48, 49] while no amplification was observed in the symmetric sample S-DGG [49].

The drain-voltage-dependent transition from absorption with redshift to amplification with blue shift via transparent regime was investigated in two ways based on plasmonic instabilities [49] and non-plasmonic instabilities [48]. Both models qualitatively well support/reproduce the observed GDPP responses and their transition from absorption to amplification with increasing *V*_d_. If one assumes the occurrence of the GDP instability the most probable mechanism would be the TTM type instability [49]. The new modeling opens a pathway to interpret the plasmonic amplification without involving instability physics [48]. Each of these models, however, still remains small quantitative discrepancies, the former between the measured and theoretically predicted drift and plasmon velocities versus applied *V*_d_ and *V*_g_ voltages [48, 49] whereas the latter between the measured and theoretically predicted resonant mode frequencies versus applied *V*_d_ and *V*_g_ voltages [48]. The quantitative understanding of these discrepancies is a topic of future studies.

The plasmonic instabilities in 2DESs are a reliable method of the THz light generation in contrast to optical down-conversion to THz frequencies based on nonlinear optical effects and the optical-to-THz conversion through photoconduction. The latter presents a very low conversion efficiency and the former also is inherently inefficient due to the optical and THz phase mismatch limiting the efficient field interaction length. Our new phenomenological device modeling suggested a possibility of a plasmonic amplification mechanism without the need for instabilities [48]. Moreover, while another type of emitters such as quantum-cascade lasers operates exclusively at cryogenic temperatures, all results presented in this work were obtained at room temperatures.

## 4 Controlling the PT symmetry of GDP metasurfaces for ultrafast gain switching

### 4.1 Tuning the GDPP gain/loss properties by controlling the singularity points for the PT symmetry

**Figure 10:**
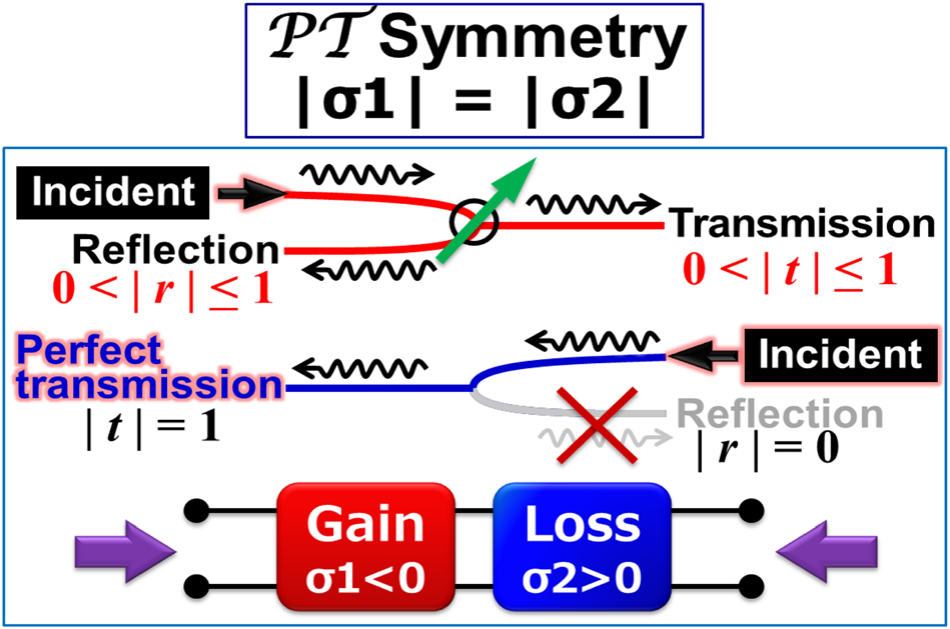
Expression of a PT-symmetric system and its anisotropic electromagnetic wave propagation.

In 1998 a new discovery was made in quantum mechanics [50]. That is, even if the system is non-Hermitian, real solutions are obtained if it preserves the parity and time reversal symmetries (we call hereafter PT symmetry). The parity symmetry means the spatial inversion symmetry so that the linear operator P transforms coordinates ( r↔−r , and k↔−k **)** and momenta (p↔−p) where **r** is a position vector, **k** is a wave vector, and **p** is a momentum. On the other hand, the time reversal symmetry is the temporal inversion symmetry so that the linear operator T performs the temporal inversion (t↔−t) , thus the frequency inversion (ω↔−ω) . The angular frequency *ω* in a substance is characterized by the complex permittivity $\epsilon^{*}=\epsilon_{real}+j\epsilon_{im}$ whose imaginary part $\epsilon_{im}$ is given by the conductivity σ :

$$\omega =kc/\sqrt{\epsilon_{real}+j\sigma /\omega }$$

where *k* is the wave number, *c* is the speed of light in vacuum. Thus, the time reversal symmetry is given by σ↔−σ . This means that the PT-symmetric system must be non-adiabatic and allows energy to flow in and out [50]. Since the positive (negative) conductivity gives loss (gain) to the system, the PT symmetry is expressed by a pair of complementary gain and loss elements (see Figure 10). This gain-loss balance leads to the existence of exceptional points at the real frequency axis in the exact PT-phase, resulting in the extraordinary frequency response of spectral singularity like the other great discovery of ‘unidirectionality’ [51]. It means that the electromagnetic wave propagation becomes anisotropic and unidirectional; the incident from the gain port gives normal transmission/reflection whereas the incident from the loss port gives a perfect transmission without reflection [51]. This extraordinary anisotropic wave propagation behavior has opened a new paradigm in laser physics like active control of the laser cavity *Q* values in various materials [52] including graphene metamaterials [53], [54], [55].

**Figure 11:**
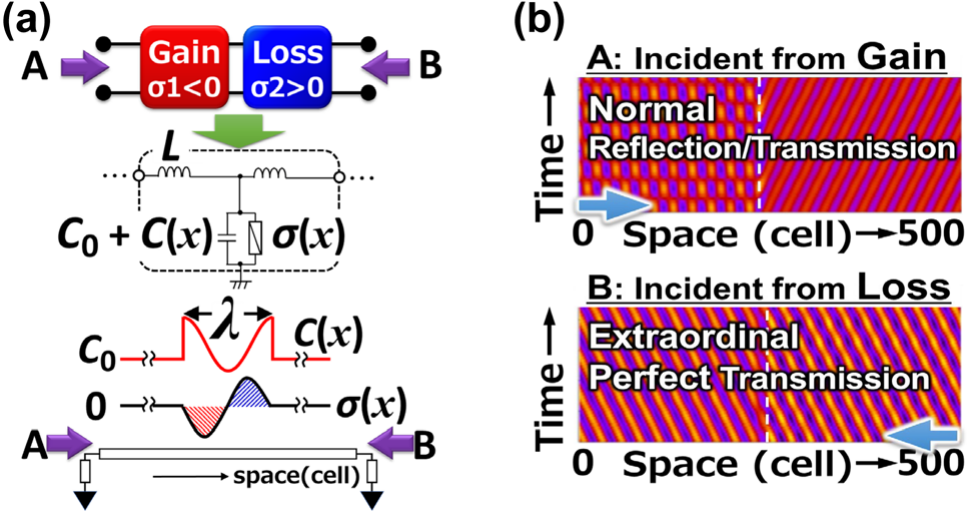
Anisotropic wave propagation in a PT-symmetric system demonstrating the perfect unidirectionality. (a) A simple toy model of a PT-symmetric transmission line consisting of a series of the unit kinetic-inductance/electrostatic-capacitance/Drude-conductance circuitry with only the central unit cell giving a nonzero sinusoidal complementary gain/loss function for its capacitance and conductance element. (b) Numerically simulated wave propagation incident from the gain port (upper) and from the loss port (lower). The spatial distribution of the phase of the wave along the horizontal axis is temporally developed along the vertical axis. Normal reflection/transmission propagation gives cross-hatched pattern whereas perfect transmission gives an angled mono-stripe pattern.

Such an anisotropic wave propagation in a PT-symmetric plasmonic metasurface can be numerically reproduced by using a simple toy model of a transmission line consisting of a series of the unit kinetic-inductance/electrostatic-capacitance/Drude-conductance circuitry as shown in Figure 11(a). The wave propagation was numerically simulated for the 500 consecutive cells with only the central unit cell giving a nonzero and sinusoidal complementary gain/loss function for its capacitance and conductance as shown in Figure 11(a). Figure 11(b) clearly reproduces the normal reflection/transmission for the incident from the gain port, and extraordinary perfect transmission for the incident from the loss port.

**Figure 12:**
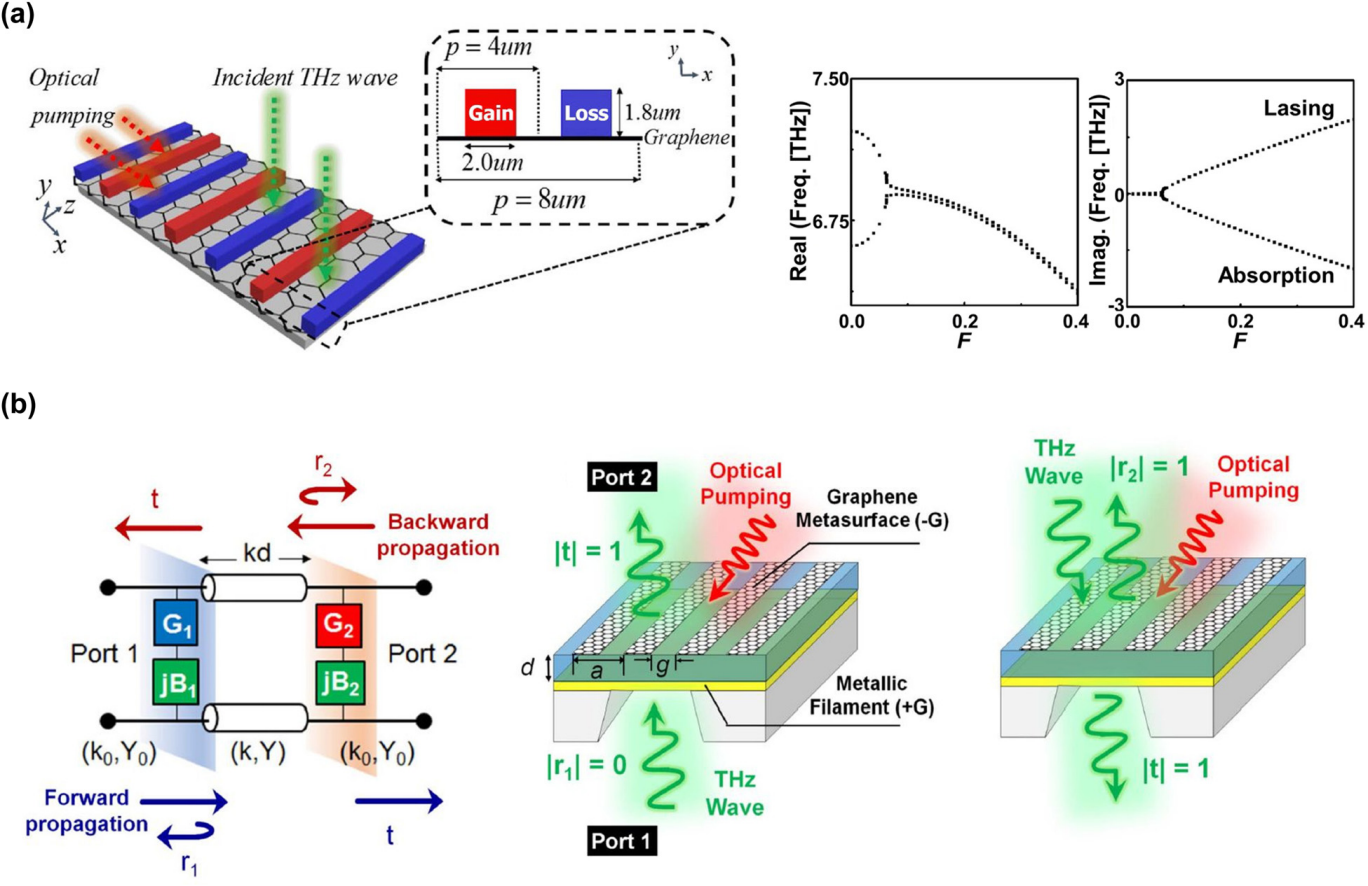
Proposed ideas for optically-pumped graphene-based PT systems controlling the unidirectionality of the THz radiation. (a) Monolayer graphene and PT-diffraction grating composite metasurface and calculated complex dispersion curves of the eigen frequencies (*k*_*x*_ = 0) as a function of the non-Hermiticity coefficient *F*. Adapted with permission from ref. [53]. Copyright 2017, the Authors, published by the Nature Publishing Group. (b) A vertically PT-symmetric graphene metasurface consisting of graphene nanoribbon grating stripes under optical pumping. A metallic filament layer working as a lossy layer is placed beneath the graphene metasurface. The system responds to be unidirectional to the vertical radiation incidence from the bottom side whereas it does to be normal reflection/transmission to that from the top side. The spectral singularity point that gives the PT symmetry can be tuned by the pumping conditions. Adapted with permission from ref. [54]. Copyright 2017, IOP Publishing Ltd. and Deutsche Physikalische Gesellschaft.

So far, several different structures of optically-pumped graphene-based PT systems manipulating their spectral singularities have been proposed and their optical gain/loss modes of frequency tunability have been numerically demonstrated (see Figure 12(a) and (b)) [53], [54], [55]. Figure 12(a) depicts a monolayer graphene and PT-diffraction grating composite metasurface. Optical pumping enables periodically localized gain/loss section in graphene reflecting the diffraction grating to make the whole structure to be PT-symmetric. The pump-dependent gain/loss characteristics of the surface conductivity in graphene tunes the spectral singularity points of the lasing threshold [53]. One can characterize the non-Hermiticity of the system by introducing the non-Hermiticity coefficients *F*_gain_ and *F*_loss_ as $\epsilon =\epsilon_{real}\left(1\mp jF_{gain/loss}\right)$ . The PT symmetry is held only when $F_{gain}=F_{loss}\equiv F$ . The real and imaginary part of the eigenfrequency Re: *f*_eigen_ and Im: *f*_eigen_ (*k*_*x*_ = 0) take peculiar route loci as a function of *F* as shown in Figure 12(a). When Re: *f*_eigen_ values coalesce (at F≳ 0.0626 ) the PT symmetry is broken, and the gain (lasing) and loss (absorption) modes coexist as their Im: *f*_eigen_ values get separated. Depending on the competition between the gain and loss mode the system works as an amplifier or absorber [53].

Figure 12(b) depicts a different structure of the vertically PT-symmetric graphene metasurface consisting of graphene nanoribbon grating stripes under optical pumping [54]. A metallic filament layer working as a lossy layer is placed beneath the graphene metasurface. The system responds to be unidirectional to the vertical radiation incidence from the bottom side whereas it does to be normal reflection/transmission to that from the top side. The spectral singularity point that gives the PT-broken symmetry can be tuned by the pumping conditions [54].

For both cases, the systems under consideration are based on ‘optical pumping’ and the wave propagation geometries perpendicular to the graphene surface. A new approach based on ‘current-injection electrical pumping’ and wave propagation geometries parallel to the graphene surface allows us to explore practical, low-power consumption, integrated planar device technologies, which will be disclosed in the next subsection.

### 4.2 Implementation of the PT-symmetric systems in the DGG-GDPP metasurfaces

**Figure 13:**
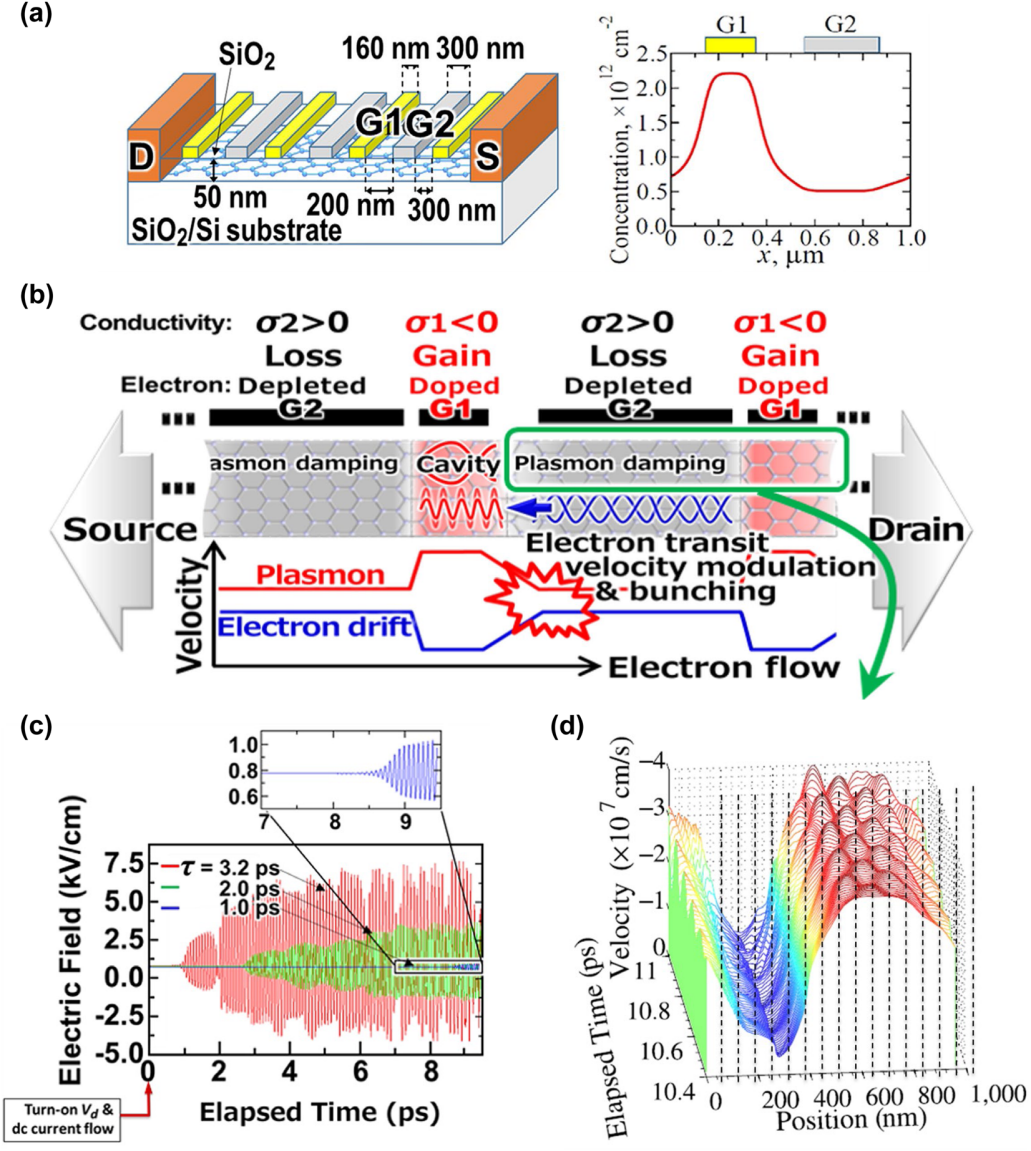
An idea and impact for implementation of the PT-symmetric system in a DGG-GDPP metasurface. (a) An asymmetric DGG-GFET structure (left) and periodic doping (right). (b) A possible scenario to promote the TTM-type GDP instability in the DGG-GFET metasurface. (c) Numerically simulated temporal evolutions of the longitudinal electric field intensity in the GDP cavity for different carrier momentum relaxation times when the drain bias voltage *V*_d_ is turned on at time zero. (d) Numerically simulated time-varying spatial distribution of the longitudinal electric field intensity in the unit PT-symmetric cell in the GDP cavity.

Implementation of the PT-symmetric systems in the DGG-GDPP metasurfaces may open a new pathway toward ultrafast gain-switch modulation of graphene-based current-injection plasmonic THz laser transistors as a practical, low-power consumption, integrated device technology. Consider an asymmetric DGG-GFET metasurface structure as described in Section 3.3 (see Figure 13(a)). One can expect the promotion of GDPP instability of the DS-, TTM-, CK-, and/or PB-types. Figure 13(b) schematically draws a possible scenario to promote the TTM-type instability. By complementary applying the DGG gate G1 and G2 voltages the highly doped graphene region underneath G1 works for a GDPP cavity whereas the depleted region underneath G2 serves an electron transit region. Under a dc drain-source bias condition with a channel dc current flow, the TTM-type instability can be promoted, resulting in self-oscillation of the GDPP radiation emission at the plasmon mode frequency. As seen in Figure 13(b) the GDPP metasurface forms a periodic arrangement of the unit cells incorporating pairs of the gain and loss regions when the GDP instability is promoted. The PT symmetry can be controlled (to be held or to be broken) by altering the gate and/or drain bias voltage.

We numerically analyzed how fast the laser cavity *Q* values can be dynamically controllable in the DGG GFET by using self-consistent simulation based on the quasi-classical Boltzmann equation [70]. The structure and dimensions that we assumed are shown in Figure 13(a). The control parameter is the drain bias voltage *V*_d_ to be turned on at time zero. The electric field intensity, which is mainly applied to the depleted electron-transit region, was set at 0.8 kV/cm. The equivalent electron velocity is $4\times 10^{7}$ cm/s at the field-effect mobility of 50,000 cm^2^/Vs. Figure 13(c) plots the temporal evolutions of the longitudinal electric field in the GDP cavity for different carrier momentum relaxation times τ = 1.0, 2.0, and 3.2 ps. Originally the graphene metasurface with zero-*V*_d_ is entirely lossy. With increasing the *V*_d_ and the level of current injection pumping the GDP instability started to be promoted. When the instability-driven gain balances to the loss in the depleted region, the system becomes PT-symmetric. A further increase of *V*_d_ makes the level of instability-driven gain surpass that of the loss, resulting in self-oscillations of the radiation emission. The important feature is its ultrafast transition speed within 10 ps duration. Superior property with a longer τ value exhibits faster turning on with stronger field intensity of ∼9.5 THz self-oscillation (identified by the Fourier transformed spectra). Even the materials of a rather limited quality with a τ value of 1 ps still show a fast transition to the stationary stage oscillation within a 10 ps duration corresponding to the single-bit time slot for the 100 Gbit/s data rate. Figure 13(d) plots numerically simulated time-varying spatial distribution of the longitudinal electric field intensity in the unit PT-symmetric cell in the GDP cavity. The red-colored high-velocity electrons are confined in the gain section whereas the blue-colored low-velocity electrons stay in the loss section. These results demonstrate that a 100 Gbit/s-class ultrafast data coding for use in 6G and 7G THz wireless communication systems is feasible by our proposed technology.

### 4.3 Toward graphene plasmonic THz laser transistors

**Figure 14:**
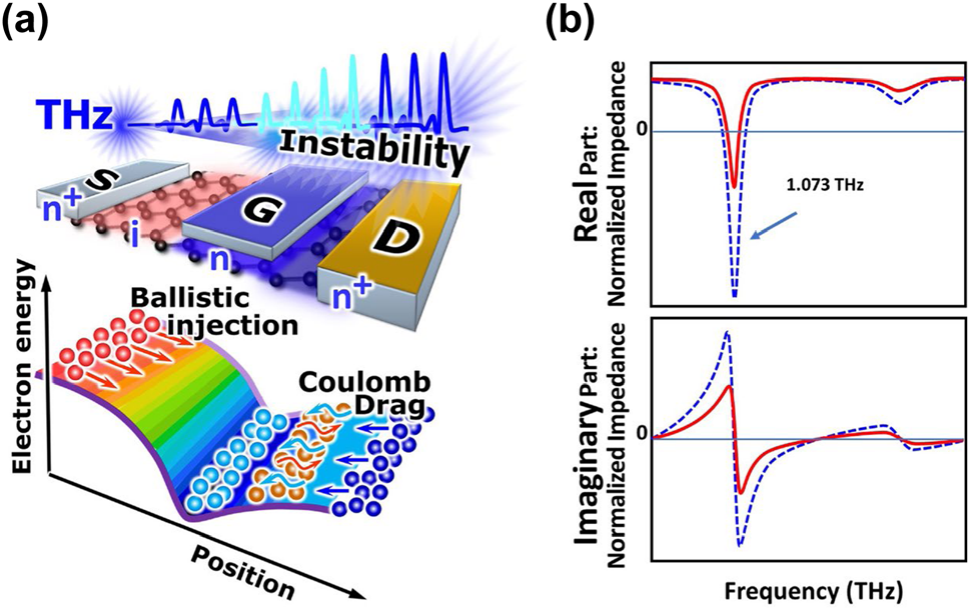
The Coulomb drag effect in a lateral n^+^–i–n–n^+^ GFET structure with ballistic injection of graphene Dirac fermions makes it possible to strongly modify the current-voltage characteristics to produce ‘gain’ in the terahertz frequency range, leading to current-driven GDP instability and self-oscillatory generation of THz coherent radiation.(a) The band diagram illustrating the case when the gate is positively biased to make an n-type doping in the gated channel region and the drain is modestly biased so as to obtain an inverted potential slope between the gated channel and the drain side. The Coulomb drag effect promotes the reverse injection of quasi-equilibrated electrons from the drain side. Adapted with permission from ref. [72]. Copyright 2022, Wiley-VCH GmbH. (b) Numerically simulated dynamic conductivities demonstrating the resonant negative dynamic conductivity. The solid and dashed lines are for moderate and strong drag effect cases, respectively. Adapted with permission from ref. [72]. Copyright 2022, Wiley-VCH GmbH.

Recently, a new GDP instability mechanism associated with the injection of high energy ballistic electrons into the quasi-equilibrium plasma and their Coulomb drag in n^+^–i–n–n^+^ GFETs and similar devices has been brought forward [71, 72] as depicted in Figure 14 [72]. In principle, the Coulomb drag mechanism might be relevant to the plasma wave generation in the periodic GFET structures analogous to the previously considered two-stream instability systems [73] with ballistic carrier flows and quasi-equilibrated non-traversing carriers. What makes a critical difference and advantage in the newly proposed system in the GFETs compared to the previous implementations is (i) an extraordinary transport property of GDFs enabling ballistic transport over micrometers in the graphene channel even at room temperatures and (ii) strong Coulomb drag effect mediated by the strong Coulomb intercarrier scattering of GDFs. Analytical device modeling revealed such unique properties strongly modify the channel current flow to be complex with negatively down-streaming drag current against the normal forward current under a certain drain-source potential region, resulting in negative differential conductance [72, 74] and promotion of the GDP instability with THz self-oscillation in a THz frequency region in standard micrometer-size GFETs (see Figure 14).

**Figure 15:**
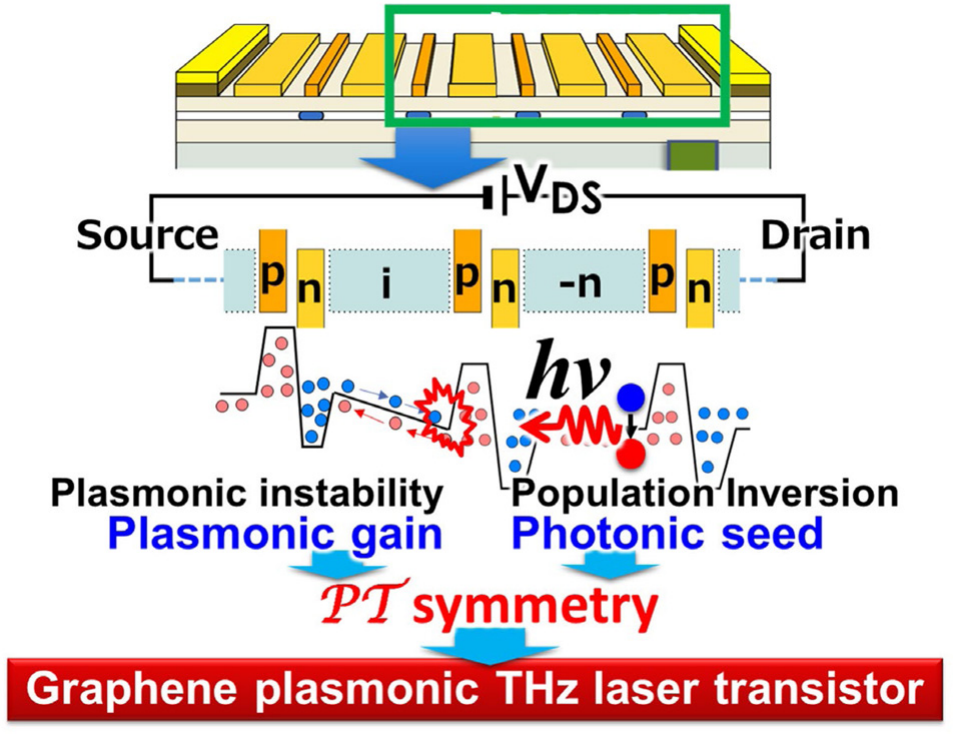
A possible structure for graphene plasmonic THz laser transistor consisting of a series of unit sections including the photonic seed and plasmonic gain sections.

Based on the aforementioned discussions, an integration of these newly obtained knowledges may enable a possible design of the graphene plasmonic THz laser transistor based on the DGG-GFET structure shown in Figure 15. Suppose the DGG G1 and G2 are complementary biased to make bipolar (electron and hole) injections and the drain-source is forward biased. The proposed device consists of a series of unit sections including the photonic seed and plasmonic gain sections. The photonic seed section generates spontaneous THz photon emission under current-injection pumping whereas the plasmonic gain section amplifies the spontaneous THz photons mediated by the GDP instability. As is discussed in Section 4.2, an idea for controlling the PT symmetry of the DGG-GFET metasurface help improve the laser device performance and functionalities like ultrafast gain-switching modulation.

The photonic seed section can be configured by selective weak doping (denoted with ‘–n’ in Figure 15) to the wider ungated spacing in the photonic seed section to keep near-flat band and hold the inverted carriers in it under a rather strong drain bias voltages that promote the GDP instability in the plasmonic gain section. The selective weak –n doping might be technologically challenging. Electrostatic doping using regional back-gate biasing is a mature solution but is not easy for epitaxial graphene thermally decomposed on a SiC substrate due to the need for ultra-thinning the semi-insulating SiC substrate. Chemical doping is another solution but is suffering from unintentional doping to the other portions and/or degradation of the crystallographic quality and resultant shortening of carrier momentum relaxation time. Recently, a novel technique to synthesize high-quality few-layer epitaxial graphene on a single-crystalline SiC thin film grown on a Si wafer has been developed [75]. The precise control of the epitaxially grown graphene layers is also a remaining issue, which could be managed by introducing a microfabricating SiC substrate technique that could spatially confine the epitaxy area [76]. Novel ideas to explore the advanced device/process technology are topics of future studies.

As the final discussion of this article, we present the benchmarking of the performance projections of typical THz solid-state emitter devices depicted in Section 1: QCLs, DFG-QCLs, RTDs, and graphene-plasmonic laser transistors (GPLTs). Details of the benchmarking are summarized in Table S1 of Supplementary Material. The DFG-QCLs resolve some critical issues but suffer from low wall-plug efficiency due to material-dependent nonlinear susceptibility. The RTDs assure room-temperature operation but their operating frequencies are limited below 2.5 THz with poor output power. The GPLTs are expected to offer wider operating frequencies from ∼1 to ∼10 THz with practically acceptable mW-class output power and excellent wall-plug efficiency as the crystal quality and device-process maturity improve.

## 5 Conclusions

The authors reviewed the recent advances and trends in the research and development of graphene-based plasmonic metamaterials for terahertz (THz) laser transistors. The authors’ theoretical discovery on THz laser transistors in 2007 was realized as a distributed-feedback dual-gate graphene-channel field-effect transistor (DFB-DG-GFET) in 2018, but it suffered from poor output power and rather low lasing threshold temperature. To realize room-temperature, dry-cell-battery operating intense THz lasing with fast direct modulation, various approaches based on graphene plasmonic metamaterials were introduced and discussed as real device implementations. The proposed designs include (i) replacement of the laser photonic cavity with plasmonic cavity enormously improving the THz photon field confinement with larger gain overlapping, (ii) introduction of THz amplification of stimulated emission via current-driven graphene Dirac plasmons (GDPs), and (iii) controlling the parity and time-reversal symmetry of GDPs enabling ultrafast direct gain-switch modulation. Possible real device structures and design constraints were discussed as a pathway toward coherent light sources applicable to future 6G- and 7G-class THz wireless communication systems. Benchmarking the performance projections of various practical solid-state THz emitter devices showed that the graphene-based plasmonic laser transistors will be a promising candidate for the coherent light sources of the future THz wireless communication systems. The graphene plasmonic metamaterial solutions presented here offer new ways for designing efficient devices for future robust far-infrared and THz plasmonic device technology and establish new important challenges for theoretical physics. New physical models should provide a full quantitative description of current-driven plasma phenomena in graphene and other 2D systems with Dirac-like energy band structure.

## Supplementary Material

Supplementary Material Details

## References

[1] Tonouchi M. (2007). Cutting-edge terahertz technology. *Nat. Photon.*.

[2] Chattopadhyay G. (2001). Technology, capabilities, and performance of low power terahertz sources. *IEEE Trans. THz Sci. Technol.*.

[3] Dhillon S. S., Vitiello M. S., Linfield E. H. (2017). The 2017 terahertz science and technology roadmap. *J. Phys. D Appl. Phys.*.

[4] Sengupta K., Nagastsuma T., Mittleman D. M. (2018). Terahertz integrated electronic and hybrid electronic-photonic systems. *Nat. Photon.*.

[5] Khalatpour A., Paulsen A. K., Deimert C., Wasilewski Z. R., Hu Q. (2021). High-power portable terahertz laser systems. *Nat. Photon.*.

[6] Hu B. B., Nuss M. C. (1995). Imaging with terahertz waves. *Opt. Lett.*.

[7] Mittleman D. M., Gupta M., Neelamani R., Baraniuk R. G., Rudd J. V., Koch M. (1999). Recent advances in terahertz imaging. *Appl. Phys. B Laser Opt.*.

[8] Serita K., Mizuno S., Murakami H. (2012). Scanning laser terahertz near-field imaging system. *Opt. Express*.

[9] Redo-Sanchez A., Heshmat B., Aghasi A. (2016). Terahertz time-gated spectral imaging for content extraction through layered structures. *Nat. Commun.*.

[10] Mittleman D. M. (2018). Twenty years of terahertz imaging. *Opt. Express*.

[11] Yu C., Fan S., Sun Y., Pickwell-Macpherson E. (2012). The potential of terahertz imaging for cancer diagnosis: a review of investigations to date. *Quant. Imag. Med. Surg.*.

[12] Yang X., Zhao X., Yang K. (2016). Biomedical applications of terahertz spectroscopy and imaging. *Trends Biotechnol.*.

[13] Sun Q. S., He Y. Z., Liu K., Fan S. T., Parrott E. P. J., Pickwell-MacPherson E. (2017). Recent advances in terahertz technology for biomedical applications. *Quant. Imag. Med. Surg.*.

[14] Koening S., Lopez-Diaz D., Antes J. (2013). Wireless sub-THz communication system with high data rate. *Nat. Photon.*.

[15] Nagatsuma T., Ducournau G., Renaud C. C. (2016). Advances in terahertz communications accelerated by photonics. *Nat. Photon.*.

[16] Akyildiz I. F., Kak A., Nie S. (2020). 6G and beyond: the future of wireless communications systems. *IEEE Access*.

[17] Kazarinov R. F., Suris R. A. (1971). Possibility of the amplification of electromagnetic waves in a semiconductor with a superlattice | BibSonomy. *Sov. Phys. Semiconduct.*.

[18] Faist J., Capasso F., Sivco D. L., Sirtori C., Hutchinson A. L., Cho A. Y. (1994). Quantum cascade laser. *Science*.

[19] Köhler R., Tredicucci A., Beltram F. (2002). Terahertz semiconductor-heterostructure laser. *Nature*.

[20] Williams B. S. (2007). Terahertz quantum-cascade lasers. *Nat. Photon.*.

[21] Chassagneux Y., Wang Q. J., Khanna S. P. (2012). Limiting factors to the temperature performance of THz quantum cascade lasers based on the resonant-phonon depopulation scheme. *IEEE Trans. THz Sci. Technol.*.

[22] Vitiello M. S., Scalari G., Williams B., De Natale P. (2015). Quantum cascade lasers: 20 years of challenges. *Opt. Express*.

[23(a)] Fujita K., Jung S., Jiang Y. (2018). Recent progress in terahertz difference-frequency quantum cascade laser sources. *Nanophotonics*.

[b] Fujita K., Hayashi S., Ito A., Hitaka M., Dougakiuchi T. (2019). Sub-terahertz and terahertz generation in long-wavelength quantum cascade lasers. *Nanophotonics*.

[24] Maekawa T., Kanaya H., Suzuki S., Asada M. (2016). Oscillation up to 1.92 THz in resonant tunneling diode by reduced conduction loss. *Appl. Phys. Express*.

[25] Bezhoko M., Suzuki S., Asada M. (2021). Analysis of output power characteristics for resonant-tunneling diode terahertz oscillator with cylindrical cavity resonator. *Jpn. J. Appl. Phys.*.

[26] Novoselov K. S., Geim A. K., Morozov S. V. (2004). Electric field effect in atomically thin carbon films. *Science*.

[27] Geim A. K., Novoselov K. S. (2007). The rise of graphene. *Nat. Mater.*.

[28] Grigorenko A. N., Polini M., Novoselov K. S. (2012). Graphene plasmonics. *Nat. Photon.*.

[29] Hartmann R. R., Kono J., Portnoi M. E. (2014). Terahertz science and technology of carbon nanomaterials. *Nanotechnology*.

[30] Tredicucci A., Vitiello M. S. (2014). Device concepts for graphene-based terahertz photonics. *IEEE J. Sel. Top. Quant. Electron.*.

[31] Li Y., Tantiwanichapan K., Swan A. K., Paiella R. (2020). Graphene plasmonic devices for terahertz optoelectronics. *Nanophotonics*.

[32] Ryzhii V., Ryzhii M., Otsuji T. (2007). Negative dynamic conductivity in optically pumped graphene. *J. Appl. Phys.*.

[33] Ryzhii M., Ryzhii V. (2007). Injection and population inversion in electrically induced p-n junction in graphene with split gates. *Jpn. J. Appl. Phys.*.

[34] Otsuji T., Boubanga-Tombet S., Satou A., Ryzhii M., Ryzhii V. (2013). Terahertz-wave generation using graphene-toward new types of terahertz lasers. *IEEE J. Sel. Top. Quant. Electron.*.

[35] Ryzhii V., Ryzhii M., Mitin V., Otsuji T. (2011). Toward the creation of terahertz graphene injection laser. *J. Appl. Phys.*.

[36] Satou A., Ryzhii V., Kurita Y., Otsuji T. (2013). Threshold of terahertz population inversion and negative dynamic conductivity in graphene under pulse photoexcitation. *J. Appl. Phys.*.

[37] Yadav D., Tamamushi G., Watanabe T. (2018). Terahertz light- emitting graphene-channel transistor toward single-mode lasing. *Nanophotonics*.

[38] Ryzhii V., Dubinov A. A., Otsuji T., Mitin V., Shur M. S. (2010). Terahertz lasers based on optically pumped multiple graphene structures with slot-line and dielectric waveguides. *J. Appl. Phys.*.

[39] Popov V. V., Polischuk O. V., Davoyan A. R., Ryzhii V., Otsuji T., Shur M. S. (2012). Plasmonic terahertz lasing in an array of graphene nanocavities. *Phys. Rev. B*.

[40] Popov V. V., Polischuk O. V., Nikitov S. A., Ryzhii V., Otsuji T. (2013). Amplification and lasing of terahertz radiation by plasmons in graphene with a planar distributed Bragg resonator. *J. Opt.*.

[41] Dubinov A. A., Aleshkin V., Morozov S. V., Ryzhii V., Otsuji T. (2019). Terahertz plasmon-emitting graphene-channel transistor. *Opto-Electron. Rev.*.

[42] Dubinov A. A., Aleshkin V., Mitin V., Otsuji T., Ryzhii V. (2011). Terahertz surface plasmons in optically pumped graphene structures. *J. Phys. Condens. Matter*.

[43] Watanabe T., Fukushima T., Yabe Y. (2013). The gain enhancement effect of surface plasmon polaritons on terahertz stimulated emission in optically pumped monolayer graphene. *New J. Phys.*.

[44] Page A. F., Ballout F., Hess O., Hamm J. M. (2015). Nonequilibrium plasmons with gain in graphene. *Phys. Rev. B*.

[45] Polischuk O. V., Fateev D. V., Otsuji T., Popov V. V. (2017). Plasmonic amplification of terahertz radiation in a periodic graphene structure with the carrier injection. *Appl. Phys. Lett.*.

[46] Tarasenko I. I., Page A. F., Hamm J. M., Hess O. (2019). Nonlocal quantum gain facilitates loss compensation and plasmon amplification in graphene hyperbolic metamaterials. *Phys. Rev. B*.

[47] Morgado T. A., Silveirinha M. G. (2021). Active graphene plasmonics with a drift-current bias. *ACS Photonics*.

[48] Boubanga-Tombet S., Knap W., Yadav D. (2020). Room temperature amplification of terahertz radiation by grating-gate graphene structures. *Phys. Rev. X*.

[49] Boubanga-Tombet S., Satou A., Yadav D. (2021). Paving the way for tunable graphene plasmonic THz amplifiers. *Front. Phys.*.

[50] Bender C. M., Boettcher S. (1998). Real spectra in non-Hermitian Hamiltonians having PT symmetry. *Phys. Rev. Lett.*.

[51] Ramezani H., Kottos T. (2010). Unidirectional nonlinear PT-symmetric optical structures. *Phys. Rev. A*.

[52(a)] Miri M.-A., Alu A. (2019). Exceptional points in optics and photonics. *Science*.

[b] Krasnok A., Alu A. (2020). Active nanophotonics. *Proc. IEEE*.

[53] Zhang W., Wu T., Zhang X. (2017). Tailoring eigenmodes at spectral singularities in graphene-based PT systems. *Sci. Rep.*.

[54] Sakhadari M., Farhat M., Chen P.-Y. (2017). PT-symmetric metasurfaces: wave manipulation and sensing using singular points. *New J. Phys.*.

[55] Zhernovnykova O. A., Popova O. V., Deynychenko G. V., Dynichenko T. I., Bludov Y. V. (2019). Surface plasmon-polaritons in graphene, embedded into medium with gain and losses. *J. Phys. Condens. Matter*.

[56] Krasheninnikov M., Chaplik A. (1980). Instabilities of two-dimensional plasma waves. *Sov. Phys. JETP*.

[57] Tsui D., Gornik E., Logan R. (1980). Far infrared emission from plasma oscillations of Si inversion layers. *Solid State Commun.*.

[58] Dyakonov M., Shur M. (1993). Shallow water analogy for a ballistic field effect transistor: new mechanism of plasma wave generation by dc current. *Phys. Rev. Lett.*.

[59] Mikhailov S. (1998). Plasma instability and amplification of electromagnetic waves in low- dimensional electron systems. *Phys. Rev. B*.

[60] Ryzhii V., Satou A., Shur M. S. (2006). Plasma instability and terahertz generation in HEMTs due to electron transit-time effect. *IEICE Trans. Electron.*.

[61] Aizin G., Mikalopas J., Shur M. (2016). Current-driven plasmonic boom instability in three- dimensional gated periodic ballistic nanostructures. *Phys. Rev. B*.

[62] Matov O., Meshkov O., Polischuk O., Popov V. (1997). Generation of submillimeter electro- magnetic radiation from two-dimensional plasma waves in a semiconductor heterostructure with metal grating. *Physica A*.

[63] Allen J. S., Tsui D., Logan R. (1997). Observation of the two-dimensional plasmon in silicon inversion layers. *Phys. Rev. Lett.*.

[64] Ando T., Fowler A. B., Stern F. (1982). Electronic properties of two-dimensional systems. *Rev. Mod. Phys.*.

[65] Hirakawa K., Yamanaka K., Grayson M., Tsui D. (1995). Far-infrared emission spectroscopy of hot two-dimensional plasmons in Al0.3Ga0.7As/GaAs heterojunctions. *Appl. Phys. Lett.*.

[66] Boubanga-Tombet S., Teppe F., Torres J. (2010). Room temperature coherent and voltage tunable terahertz emission from nanometer-sized field effect transistors. *Appl. Phys. Lett.*.

[67] El Fatimy A., Dyakonova N., Meziani Y. (2010). AlGaN/GaN high electron mobility transistors as a voltage-tunable room temperature terahertz source. *J. Appl. Phys.*.

[68] Knap W., Dyakonov M. (2013). *Handbook of Terahertz Technology for Imaging, Sensing and Communications*.

[69] Smith S. J., Purcell E. (1953). Visible light from localized surface charges moving across a grating. *Phys. Rev.*.

[70] Koseki Y., Ryzhii V., Otsuji T., Popov V. V., Satou A. (2016). Giant plasmon instability in a dual-grating-gate graphene field-effect transistor. *Phys. Rev. B*.

[71] Ryzhii V., Ryzhii M., Mitin V., Shur M. S., Otsuji T. (2021). Coulomb electron drag mechanism of terahertz plasma instability in n+-i-n-n+ graphene FETs with ballistic injection. *Appl. Phys. Lett.*.

[72] Ryzhii V., Ryzhii M., Satou A., Mitin V., Shur M. S., Otsuji T. (2021). Ballistic injection terahertz plasma instability in graphene n + -i-n-n + field-effect transistors and lateral diodes. *Phys. Status Solidi*.

[73] Gribnikov Z. S., Vagidov N. Z., Mitin V. V. (2000). Tow-stream instability and oscillatory regimes induced in ballistic diodes and field-effect transistors. *J. Appl. Phys.*.

[74] Ryzhii V., Ryzhii M., Mitin V., Shur M. S., Otsuji T. (2021). S-shaped current-voltage characteristics of n+-i-n-n+ graphene field-effect transistors due to the Coulomb drag of quasi-equilibrium electrons by ballistic electrons. *Phys. Rev. Appl.*.

[75] Endoh N., Akiyama S., Tashima K. (2021). High-quality few-layer graphene on single-crystalline SiC thin film grown on affordable wafer for device applications. *Nanomaterials*.

[76] Fukidome H., Kawai Y., Fromm F. (2012). Precise control of epitaxy of graphene by microfabricating SiC substrate. *Appl. Phys. Lett.*.

