# Supplementary material for: Graphene-based plasmonic metamaterial for terahertz laser transistors
Source: Nanophotonics. 2022 Feb 2;11(9):1677–96. doi: 10.1515/nanoph-2021-0651 (PMC11502014; doi:10.1515/nanoph-2021-0651)
Supplement: Supplementary file 1 — Supplementary Material Details [file j_nanoph-2021-0651_suppl.pdf]

Supplementary Material

Title:

Graphene-based plasmonic metamaterial for terahertz laser transistors

Short title:

Graphene plasmonic terahertz laser transistors

Authors:

Taiichi Otsuji[1], Stephane Albon Boubanga-Tombet[1], Akira Satou[1], Deepika Yadav[1], Hirokazu Fukidome[1], Takayuki Watanabe[1], Tetsuya Suemitsu[2], Alexander A. Dubinov[3], Vyacheslav V. Popov[4], Wojciech Knap[1,5,6], Valentin Kachorovskii[7], Koichi Narahara[8], Maxim Ryzhii[9], Vladimir Mitin[10], Michael S. Shur[11], and Victor Ryzhii[1,12]

[1] Research Institute of Electrical Communication, Tohoku University, Sendai 9808577, Japan

[2] Center for Innovative Integrated Electronic Systems, Tohoku University, Sendai 9808572, Japan

[3] Institute for Physics of Microstructures, Russian Academy of Sciences, Lobachevsky State University of Nizhny Novgorod, Nizhny Novgorod 603950, Russia

[4] Kotelnikov Institute of Radio Engineering and Electronics (Saratov Branch), Russian Academy of Sciences, Saratov 410019, Russia

[5] CENTERA Laboratories, Institute of High Pressure Physics, Warsaw PAS 01142, Poland

[6] Laboratory Charles Coulomb, University of Montpellier and CNRS, Montpellier F-34095, France

[7] Ioffe Institute, 194021 St. Petersburg, Russia

[8] Department of Electrical and Electronic Engineering, Kanagawa Institute of Technology, Atsugi, Kanagawa 243-0292, Japan

[9] Department of Computer Science and Engineering, University of Aizu, Aizu-Wakamatsu 965-8580, Japan

[10] Department of Electrical Engineering, University at Buffalo, SUNY, Buffalo, NY 14260, USA

[11] Department of Electrical, Computer, and Systems Engineering, Rensselaer Polytechnic Institute, Troy, NY 12180, USA

[12] Mokerov Institute of Ultra-High Frequency Semiconductor Electronics, RAS, Moscow 117105, Russia

S1. Benchmarking of the performance projections of typical THz solid-state emitter devices

We present the benchmarking of the performance projections of typical THz solid-state emitter devices as depicted in Section 1: QCLs, DFG-QCLs, RTDs, and graphene-plasmonic laser transistors (GPLTs). Details of the benchmarking are summarized in Table S1. Advantageous figures are shown in blue fonts, while disadvantageous figures are shown in red fonts. The QCLs suffer from phonon decoherence preventing from

room-temperature operation. The DFG-QCLs resolve some critical issues but suffer from low wall-plug efficiency due to material-dependent nonlinear susceptibility. The RTDs assure room-temperature operation but their operating frequencies are limited below 2.5 THz with poor output power. Please be reminded that the projection values for the wall-plug efficiency are not directly cited from any prior publications but are the authors' speculations based on the data disclosed in the prior publications. Nevertheless, the GPLTs are expected to offer wider operating frequencies from ~1 to ~10 THz with practically acceptable mW-class output power and excellent wall-plug efficiency as the crystal quality and device-process maturity improve.

Table S1. Benchmarking of the performance projections of typical THz solid-state emitter devices.

Figure of Merits	Obtained/ Projection	QCL [5, 21, 22]	DFB-QCL [23]	RTD [23, 24]	GPLT* [37, 48, 49]
Limiting Factors		Phonon decoherence	Nonlinear susceptibility	Electron transit time	Drude free carrier absorption
Issues		Operating temperature<br>Lower operating frequency	Spectral quality<br>Wall-plug efficiency	Upper operating frequency<br>Output power	Crystal quality<br>Process maturity
Operating Frequency	Obtained	down to 1.2 THz [21, 22]	0.7 ~ 3.0 THz	up to 1.92 THz [23]	5.2 THz [37] in single-mode lasing<br>1.0 ~ 3.0 THz [48, 49] in amplification
	Projection	down to 1.0 THz [21, 22]	down to 0.5 THz	up to 2.5 THz [24]	1.0 ~ 10 THz
Max. Operating Temperature	Obtained	up to 250 K [5] in pulsed mode	RT in c.w. mode	RT in c.w. mode	100 K [37] in c.w. mode<br>RT [48, 49] in pulsed mode
	Projection	close to RT	RT	RT	RT
Max. Output Power	Obtained	10 μW in pulsed mode<br>at 250 K at 4 THz [5]<br>20 mW in pulsed mode<br>at 240 K at 4 THz [5]	10 μW in c.w. mode<br>at RT at 1.6 THz<br>1 mW in pulsed mode<br>at RT at 1.6 THz	0.4 μW in c.w. mode<br>at RT at 1.92 THz [23]	0.1 μW in c.w. mode<br>at 100 K at 5.2 THz [37]<br>2.7 μW in pulsed mode<br>at RT at 3 THz [48, 49]
	Projection	up to 1 mW<br>at ~RT at 1 THz [22]	up to sub-mW<br>at RT at 1 THz	up to 50 μW / 0.5 μW<br>at RT at 1.5 THz / 2.5 THz [24]	up to 1 mW<br>at RT at 1 ~ 8 THz [49]
Wall-Plug Power	Obtained	4W<br>at 250 K at 4 THz [5]	~10 W<br>at RT at 1.6 THz	~50 mW<br>at RT at 1.92 THz [23]	~0.6 mW at 100 K at 5.2 THz [37]<br>~32 μW at RT at 3 THz [48, 49]
Wall-Plug Efficiency	Obtained	2.50E-06 at 250 K [5]<br>5.00E-03 at 240 K [5]	1.00E-04 at RT	1.00E-04 at RT [23]	1.00E-04 at 100 K [37]<br>~0.1 at RT in pulsed mode [49]
	Projection	1.00E-03 at 250 K<br>1.00E-05 at close to RT	1.00E-04 at RT	1.00E-03 at RT	1.00E-02 at RT

* GPLT: Graphene Plasmonic Laser Transistor
